# Evolution of duplicated *IgH *loci in Atlantic salmon, *Salmo salar*

**DOI:** 10.1186/1471-2164-11-486

**Published:** 2010-09-02

**Authors:** Motoshige Yasuike, Johan de Boer, Kristian R von Schalburg, Glenn A Cooper, Linda McKinnel, Amber Messmer, Stacy So, William S Davidson, Ben F Koop

**Affiliations:** 1 Department of Biology, University of Victoria, PO Box 3020 STN CSC, Victoria, British Columbia, V8W 3N5, Canada; 2 Department of Molecular Biology and Biochemistry, Simon Fraser University, 8888 University Drive, Burnaby, British Columbia, V5A1S6, Canada

## Abstract

**Background:**

The Atlantic salmon (*Salmo salar*) immunoglobulin heavy chain (*IgH*) locus possesses two parallel *IgH *isoloci (*IGH-A *and *IGH-B*), that are related to the genomic duplication event in the family Salmonidae. These duplicated *IgH *loci in Atlantic salmon provide a unique opportunity to examine the mechanisms of genome diversity and genome evolution of the *IgH *loci in vertebrates. In this study, we defined the structure of these loci in Atlantic salmon, and sequenced 24 bacterial artificial chromosome (BAC) clones that were assembled into the *IGH-A *(1.1 Mb) and *IGH-B *(0.9 Mb) loci. In addition, over 7,000 cDNA clones from the *IgH *variable (VH) region have been sequenced and analyzed.

**Results:**

The present study shows that the genomic organization of the duplicated *IgH *loci in Atlantic salmon differs from that in other teleosts and other vertebrates. The loci possess multiple Cτ genes upstream of the Cμ region, with three of the Cτ genes being functional. Moreover, the duplicated loci possess over 300 VH segments which could be classified into 18 families. This is the largest number of VH families currently defined in any vertebrate. There were significant structural differences between the two loci, indicating that both *IGH-A *and *-B *loci have evolved independently in the short time after the recent genome duplication approximately 60 mya.

**Conclusions:**

Our results indicate that the duplication of the *IgH *loci in Atlantic salmon significantly contributes to the increased diversity of the antibody repertoire, as compared with the single *IgH *locus in other vertebrates.

## Background

The adaptive immune system based on somatic recombination of immune receptor genes appeared in vertebrates some 500 million years ago (mya) [1,2]. While jawless vertebrates, such as lamprey and hagfish, assemble their variable lymphocyte receptors (VLRs) through recombination of leucine-rich repeat (LRR) modular units [3-5], jawed vertebrates generate their diverse repertoire of B and T cell antigen receptors through rearrangement of variable-(diversity)-joining (V-(D)-J) gene segments [6,7].

Immunoglobulins (Igs) are key molecules within the jawed vertebrate humoral immune system that are generated by the B cells for defence against a wide variety of pathogens. Igs are composed of two heavy (H) chains and two light (L) chains that are encoded by the *IgH *locus and *IgL *locus, respectively. Two different types of genomic rearrangement of the *IgH *locus have evolved. In cartilaginous fishes, such as sharks and skates, closely linked individual clusters of VH-D-D-J-Constant (CH) gene segments are repeated 100 - 200 times [8,9]. In contrast, in most bony vertebrates (from teleost fishes to mammals), the VH, D, JH, and CH gene segments are in tandem arrays, also known as translocon organisation (VH)_n_-(D)_m_-(JH)_x_-(CH)_y _[10-13]. The contribution of multiple germ line VH, D and JH gene segments to antibody diversity is magnified by the random rearrangement of these segments in somatic cells [14]. In response to an antigen, mature B cells can change their expressed CH region genes, and the different CH region genes that possess different effector functions. In mammals, there are five Ig classes, named for their CH region component as IgM (μ chain), IgD (δ chain), IgG (γ chain), IgA (α chain) and IgE (ε chain) [9,15]. In teleosts, the predominant serum antibody is IgM, which was the first Ig class identified [13]. Subsequently, IgD was also found, but the teleost IgD gene is expressed as a chimeric transcript that includes the first exon of the IgM gene (Cμ_1_) [16-20]. It has long been believed that teleost fish possess only IgM and IgD. However, novel Ig classes (IgTs) have recently been found in zebrafish (*Danio rerio*) (IgZ) [21], rainbow trout (*Oncorhynchus mykiss*) [22], fugu (*Fugu rubripes*) (novel IgH) [23] and carp (*Cyprinus carpio*) (IgM-IgZ chimera) [24] as more sequences have become available.

In the zebrafish and rainbow trout *IgH *locus, a CH gene (Cτ) of the novel Ig class (IgT) exists upstream of Cμ and Cδ genes, possessing its own D and JH segments [21,22]. This organization of genes in the zebrafish and rainbow trout *IgH *loci resembles the mouse T cell receptor (TCR) *α/δ *locus (*TRA/TRD*) [21]. Similarly, the fugu ortholog of IgT (novel IgH) is also found upstream of Cμ and Cδ genes and this Ig has its own D and JH segments, but the gene organization of the fugu IgT differs significantly from zebrafish and rainbow trout IgTs [23]. However, in catfish, a CH region upstream of Cμ and Cδ genes similar to IgT has not been found [25]. In addition, the catfish *IgH *locus contains three linked pairs of Cμ and Cδ genes, but only one Cμ and possibly three Cδ genes are functional [25-27]. The three different Cδ gene regions encode heavy chains of membrane and secreted IgD, and although secreted IgD has so far been found only in catfish, it may not contain a functional V-region [26]. Recently, it has been reported that the stickleback (*Gasterosteus aculeatus*) *IgH *locus contains three tandem duplicated Cτ, Cμ and Cδ genes separated by VH, D, and JH segments, as well as a fourth Cτ gene in the 3` end of the locus [28,29]. These findings indicate that there is a large amount of variability within the *IgH *loci among teleosts.

One interesting feature of the Atlantic salmon (*Salmo salar*) *IgH *locus is that it possesses two parallel *IgH *isoloci (*IGH-A *and *IGH-B*) [12,30-32], that are related to the tetraploid ancestry of the family Salmonidae [30,33]. A recent study by Shiina et al. (2005) estimated the duplication event to have taken place approx. 60 mya based on sequence divergence of duplicated MHC class I regions of rainbow trout [34] Recently, the presence of two *IgH *loci have also been demonstrated in rainbow trout by *in situ *hybridization to rainbow trout chromosomal spreads with *IgH*-positive BAC clones [22]. However, only approximately 100 kb of the 3' end of one rainbow trout *IgH *locus has been sequenced to date. Two IgM isotypes were found in Atlantic salmon and brown trout (*Salmo trutta*), while it has been suggested through gel filtration analysis that rainbow trout and Arctic char (*Salvelinus alpinus*) possess a single IgM [35]. Moreover, only one Cμ cDNA has been found from a single homozygous rainbow trout, whereas duplicated versions of the rainbow trout Cτ and Cδ genes have been suggested from cDNA variants [22]. These findings suggest that rainbow trout and Arctic char lost an intact IgM in evolution after the genera *Salmo*, *Oncorhynchus *and *Salvelinus *radiated (10 - 18 mya) [35,36]. Thus, determination of the structure of the loci in Atlantic salmon provides a unique opportunity for understanding the evolution of the *IgH *locus in salmonids.

In this study, to define the structure of the loci in Atlantic salmon, 24 bacterial artificial chromosome (BAC) clones were sequenced and complete *IGH-A *(1.1 Mb) and *IGH-B *(0.9 Mb) loci were assembled. In addition, over 7,000 clones from the *IgH *variable (VH) region cDNAs have been sequenced and analyzed. The present study shows that the Atlantic salmon *IgH *locus represents the most complex and diverse vertebrate *IgH *locus characterized to date.

## Results

### Overall organization of *IGH-A *and *IGH-B*

Two loci were assembled from overlapping BAC sequences. *IGH-A *was assembled from 7 BACs, and *IGH-B *was assembled using 8 BACs. A few contigs, internal to BACs could not be joined, resulting in two contigs for *IGH-A *and four contigs for *IGH-B*, that are separated by small gaps of unknown length in regions of repeated sequences. The sequences in the two *IgH *loci containing VH and CH regions cover approximately 670 kb (*IGH-A*) and 710 kb (*IGH-B*), respectively. Part of a second allele for *IGH-A *was identified in the assembly of a number of BAC sequences. We noted a similarity of 99.7% over contigs spanning 190 kb sequence (data not shown).

**Figure 1:**
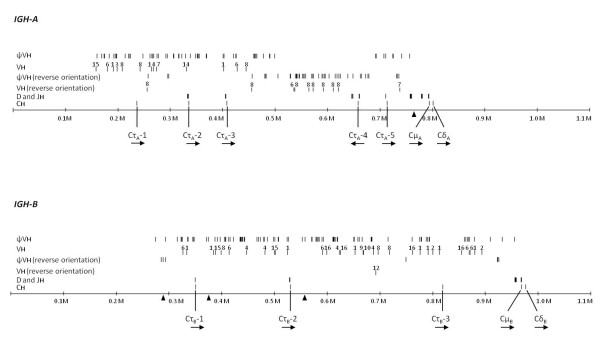
**Organization of the Atlantic salmon duplicated *IgH *loci**. The sequences in the two *IgH *loci containing VH and CH regions cover approximately 670 kb (*IGH-A*) and 710 kb (*IGH-B*), respectively. The positions of the small gaps are indicated by triangles (*IGH-A *gap at position 760.2 kb; *IGH-B *gaps at position 282.8 kb, 372.8 kb and 555.4 kb). The numbers near VH genes indicate the VH genes family numbers. Transcriptional directions for the CH genes are shown by arrowheads. A numerated version of Figure 1 is available in Additional file 1 (*IGH-A*) and Additional file 2 (*IGH-B*).

In each of the two *IgH *loci we identified numerous VH segments, many D and JH segments and several CH gene segments (Table 1, Figure 1, Additional file 1 and Additional file 2). The CH sequences are comprised of three classes, one Cμ and one Cδ in each locus and 3 and 5 complete or partial Cτ sequences, respectively. The Cμ and Cδ sequences are the most 3' elements in both loci while the constant Cτ sequences are distributed throughout the loci. D and JH sequences are generally 5' of the Cμ and Cτ sequences. Most interestingly, the region that contains the VH sequences coincides with the region that contains a large number of "Nhe I" elements, piggyBAC-like sequences that have also been recovered numerous times in the V region of the Atlantic salmon *TRA/TRD *locus [37].

**Table 1:** Summary of CH, D, JH gene segments in the duplicated loci.

CH	D	JH	in EST	comment
***IGH-A***				
τ_A_-1	-	-	no	exon 4 only
τ_A_-2	2	2	no	exon 1 and 2
τ_A_-3	0	2	no	has FS
τ_A_-4	2 (5'); 3 (3') ^a^	2	yes	reverse orientation
τ_A_-5	1	2	yes	
μ _A_	6 + 3 ^b^	5	yes	
δ _A_	-	-	yes	
***IGH-B***				
τ_B_-1	-	1	no	has FS
τ_B_-2	2	2	yes	
τ_B_-3	0	0		no exon 1
μ _B_	6	5	yes	
δ _B_	-	-	yes	

^a ^5' and 3' relative to the τ_A_-4 gene orientation, which is in reverse orientation compared to the rest of the locus.

^b ^The 3 D sequences are located approximately 20 kb 3' from the 6 D sequences and approximately 13 kb 5' of the J sequences.

The *IGH-A *and *-B *loci show 81-85% sequence identity surrounding the VH sequence region, but internally less identity than 81-85%, indicating significant reorganization involving the VH gene and NheI-elements. Additional file 3 lists identified genes flanking the loci. A dotter plot of *IGH-A *versus *IGH-B *is shown in Additional file 4. It is worth noting the similarity between the sequences flanking the two loci as this is in stark contrast to the lack of similarity between the loci themselves.

### Constant (CH) regions

**Figure 2:**
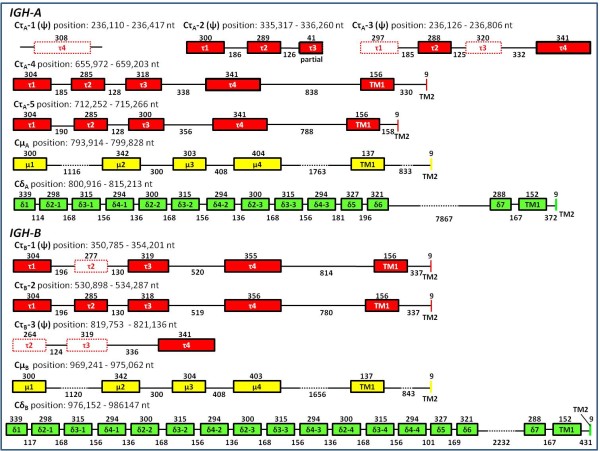
**Genomic structure of the CH and CH pseudogenes (ψ)genes (not to scale)**. The coding regions are boxes, and introns are lines. Dashed-line boxes indicate the frameshifts. The continuous regions are indicated by dotted lines. Values indicate the number of base positions.

Each locus contains several CH gene sequences, Cμ, Cδ, and Cτ. At the 3' end of each locus is one Cμ sequence followed by one Cδ sequence as previously reported [17,38]. The Cμ and Cδ sequences are approximately 98% similar between loci. Surprisingly, there are several Cτ sequences, 5 in *IGH-A *and 3 in *IGH-B*, spread out over each locus (Figure 1). The Cτ sequences in the *IGH-A *are as follows: starting from the 5' end; 1) Cτ_A_-1, partial (most of exon 4 only), 2) Cτ_A_-2, partial (5' start to approximately 40 base pairs into exon 3), 3) Cτ_A_-3, complete but has a frameshift, 4) Cτ_A_-4, complete but in reverse orientation, and 5) Cτ_A_-5, complete (Figure 2). In *IGH-B *there is 1) Cτ_B_-1, a complete sequence but with a frameshift, 2) Cτ_B_-2, complete, and 3) Cτ_B_-3, a partial (missing exon 1) (Figure 2). Cτ_B_-1 and Cτ_B_-2 are >99% identical. There are a total of two intact Cτ genes in *IGH-A *and one intact Cτ gene in *IGH-B *(Table 1 and Figure 2). The alignments of the intact CH gene amino acid sequences are available in Additional file 5. We constructed a phylogenetic tree from the translated sequences of the three intact Cτ genes and other CH genes (see Additional file 6). The three intact Cτ genes clustered within a branch containing teleost Cζ/τ sequences. Interestingly, Cτ_A _(Cτ_A_-4 and Cτ_A_-5) shared a branch with the rainbow trout Cτ, and Cτ_B_-2 branched basal to the Cτ_A_s/rainbow trout Cτ clade. It has been reported that both duplicated IgD genes in Atlantic salmon have a tandem duplication of Cδ_2_-Cδ_3_-Cδ_4 _[17,38]. However, our present study shows that the number of times exons (Cδ_2_-Cδ_3_-Cδ_4_) are repeated is different between loci, three times in *IGH-A *and four times in *IGH-B *(Figure 2). The sequence identity between these repeats of the two loci is very high (>98%), suggesting gene conversion events. The three functional Cτ genes (Cτ_B_-2, Cτ_A_-4 and Cτ_A_-5), the two Cμ genes (Cμ_A _and Cμ_B_) and Cδ genes (Cδ_A _and Cδ_B_) have been submitted to Genbank and the accession numbers are listed in Additional file 7.

### Diversity (D) segments

D gene sequences were identified through the conserved pattern of their recombination signal sequences (RSS). A total of 25 D sequence genes were identified in the two *IgH *loci (Figure 1). All segments are flanked on each end by a consensus nonamer-heptamer combination that is separated by 12 base pairs (see Additional file 8). Nine are located in the *IGH-A *5' of the Cμ gene, in a group of 6 and a group of 3. There are eight D segments associated with the three Cτ genes (0 for Cτ_A_-1 which has no 5' part; 2 for Cτ_A_-2 which has exon 1 and 2 only; 0 for Cτ_A_-3; 2 for Cτ_A_-4 with also 3 on the 3' side of the inverted Cτ_A_-4 (which is 5' in the assembly) and 1 for Cτ_A_-5) (Table 1). In *IGH-B *there are six D segments associated with the Cμ gene and two with one of the Cτ genes (Cτ_B_-2) (Table 1). Comparison of the sequences of the six D elements in each of the two groups associated with the Cμ genes in *IGH-A *and *IGH-B *indicates that the D elements occur in the same order in both loci. The additional group of three in *IGH-A *is similar to the last three of the groups of six, indicating a duplication event (see Additional file 9).

### Joining (JH) segments

Each of the two Cμ genes is preceded by five JH sequences as previously reported [12]. In addition, two JH segments are associated with each Cτ gene, except Cτ_A_-1 which has no 5' region and Cτ_B_-1 which has only a single JH segment, for a total of seven JH segments for the Cτ genes in *IGH-A *and three JH segments for the Cτ genes in *IGH-B*, located just 5' upstream of their respective Cτ gene (Table 1). All segments have a fairly conserved 5' RSS, a nonamer-heptamer combination separated by 24 base pairs (see Additional file 10). A 3' AGGT splice site is found in 18 sequences and a TGGT site in 2 sequences. A translation of the coding sequence reveals a highly conserved FDYWGKGTXVTVS amino acid sequence. One of the JH sequences (JH-τ_B_1-1) is a pseudogene as it is interrupted by a TAG stop codon.

The corresponding JH sequences in *IGH-A *and *IGH-B *are identical in coding sequence, except for JH-μ_A_-3 and JH-μ_B_-3 [12], and therefore cannot be distinguished in rearranged products.

### Variable (VH) segments

**Figure 3:**
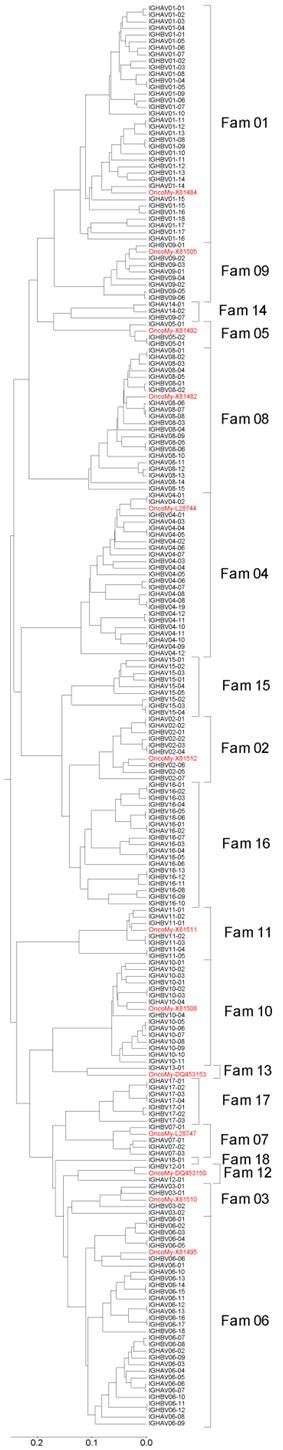
**Phylogenetic tree based on nucleotide sequences of Atlantic salmon and rainbow trout VH genes**. The tree was constructed from complete exon 2 sequences (without the RSS). These Atlantic salmon VH genes could be grouped into 18 families (Fam 01 - 18), based on >75% nucleotide similarity. Examples from thirteen VH families of rainbow trout [40,41] are shown in red letters.

Each locus contains a large number of VH genes and pseudogenes (Figure 1). A total of 153 sequences in *IGH-A *and 161 sequences in *IGH-B *were identified as matching VH gene sequences. 99 sequences in *IGH-A *and 103 sequences in *IGH-B *were characterized and named. Of these, 23 in *IGH-A *and 32 in *IGH-B* have a putative open reading frame (ORF). The deduced amino acid sequences of these VH genes have been submitted to Genbank and the accession numbers are listed in Additional file 7. The alignments of VH genes amino acid sequences are available in Additional file 11. Many other sequences are found only as fragments. VH genes start with a more or less consensus 5'-ATG(C/T)AAA(G/T)-3' octamer sequence [39] located 5' to the site of transcription initiation, and terminate at a nonamer-heptamer RSS. VH genes have a short exon 1 and a long exon 2 sequence. When complete exon 2 sequences (without the RSS) are aligned and 75% identity is applied, 18 families can be distinguished. Representative sequences from the 13 families that were identified in *Oncorhynchus mykiss *[40,41] align within 13 of these 18 families (Figure 3). The distribution of the VH families between *IGH-A *and *-B *is listed in Additional file 12. The number of sequences identified per family varies widely, from a single copy (in family 18) to 18 members (see Additional file 12). The orientation of the VH sequences indicates some rearrangement within the loci and an inversion event is evident when comparing *IGH-A *and *IGH-B *sequence (data not shown). This inversion event explains the inverse orientation of the Cτ_A_-4 sequence in *IGH-A*.

**Figure 4:**
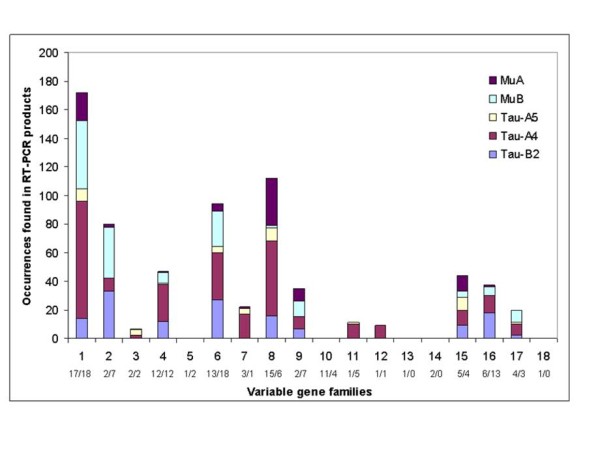
**Use of VH sequence families in rearrangements**. The numbers below the VH gene family numbers indicate distribution of VH genes in the *IGH-A*/in the *IGH-B*.

The use of VH sequences was grouped by family for analysis. VH families are used to a different extent by different constant genes. For example, family 8 is used by Cμ_A _much more frequently than by Cμ_B_, while the opposite is true for family 6 VH genes (Figure 4). The most commonly used gene families include families 1, 6, and 8, members of which comprise 60% of the putative VH ORFs. Families that contain few members with putative ORFs are also rarely recovered in ESTs.

### Rearrangements

A comprehensive set of VH specific primers (178 VH specific primers) were constructed to compliment CH specific primers and ~12 clones from all positive products sequenced to identify expression and rearrangement patterns. More than 7,000 VH-D-JH-CH cloned PCR products amplified from the kidney and spleen of two healthy individuals. We found three main types of rearrangements with VH sequences; those with a Cμ gene, those with a Cτ gene, and those with both Cμ and Cδ exons. However, not all rearrangements involving Cδ include Cμ sequence.

**Figure 5:**
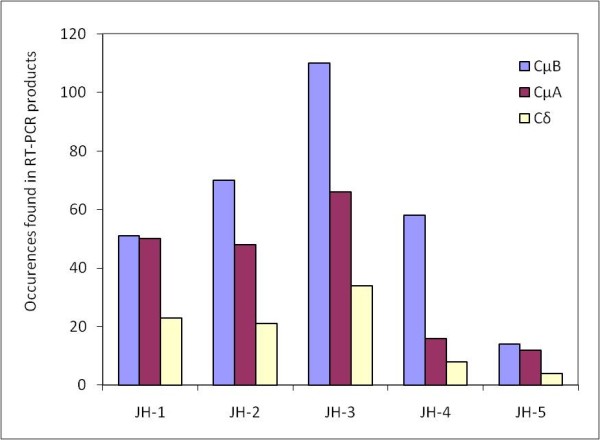
**Use of JH sequences in rearrangements**. Because many of the Cδ sequences are too similar in sequence to distinguish them unequivocally, the Cδ_A _and Cδ_B _were put into single columns.

Of 1,872 sequences generated from Cμ-specific primers, located in exon 2, 1,794 contained a conserved sequence in exon 1 of the Cμ sequence and were further analyzed. After removal of identical sequences, confirmation of an ORF in the amplified fragment, and a minimum of 98% match (BLAST) over 30 base pairs in the variable sequence, a total of 225 unique sequences were obtained containing the Cμ_A _gene and 358 sequences with the Cμ_B _gene. The JH sequences associated with the Cμ genes are not equally used; the middle JH (Cμ - JH-3) occurs most frequently in rearrangements. In fact, the use distribution for the Cμ- JH sequences by Cμ_A _and Cμ_B _is quite similar (Figure 5).

Of 1,852 sequences generated from Cτ-specific primers, 1,555 contained a conserved sequence in exon 1 of the Cτ sequence and were further analyzed. After removal of identical sequences, confirmation of an ORF in the amplified fragment, and a minimum of 98% match (BLAST) over 30 base pairs of variable sequences, a total of 140 unique sequences were obtained containing the Cτ_B_-2 gene, 284 sequences with the Cτ_A_-4 gene, and 39 sequences with the Cτ_A_-5 gene. Interestingly, these three genes are the putatively functional Cτ sequences, with the most frequently used Cτ_A_-4 gene which is in an inverted orientation. Two instances were observed where rearrangement took place with Cτ_A_-1, which has only the first 2 exons. Both occurred with VH sequences that are 5' of Cτ_A_-1. The inverted Cτ_A_-4 gene is found associated with VH sequences from both 5' and 3' of the constant sequence. In those instances where the joining sequence can be uniquely identified, we note that the Cτ_B_-2 gene is associated with JH-τ_B_1-1, rather than JH-τ_B_2-2, 95% of the time.

cDNA sequences containing Cδ were obtained with primers located in exon 3 of the Cδ sequence. There were 832 sequences of those amplified with the Cδ specific primers that contained Cδ sequences. Approximately 90% of the sequences are chimeric transcripts that contain the first exon of Cμ (Cμ_1_). However, in approximately 10% of the rearrangements involving Cδ, no Cμ_1 _sequence and generally no use of the normal splice and recombination signals is evident. A JH and VH sequence is not obvious in all of these cases in the readable sequence and in many of these rearrangements joining takes place from somewhere inside or just after the VH sequence to various distributed sites inside exon 1, 2, and 3 of the Cδ sequence (Table 2). In one recovered rearrangement, the first RSS of the Cμ-D4 sequence and the second RSS of the next Cμ-D5 sequence is used, resulting in the use of two D sequences including a 335 bp intervening genomic sequence (est 007-171). Interestingly, in many of these atypical rearrangements, joining occurs at a short repeat sequence present at both joining ends (see Table 2 for examples), indicative of a homology-directed recombination event. Because many of the Cδ sequences are too similar in sequence to distinguish them unequivocally, we were unable to distinguish the distribution of JH sequences between the Cδ_A _and Cδ_B_. However, the data still shows a preference for the middle JH by Cδ as seen in Cμ_A _and Cμ_B _sequences (Figure 5).

**Table 2:** Examples of "atypical" rearrangements involving δ.

EST ID	Sequence repeat	Rearrangement	**Join in δ**^a^
001-084	CTAG	V-(D?)-J-δ	641(e2)
002-029	CTAC	V-(D?)-J-δ	532(e2)
002-179	GANACAG	V(before RSS)^b^-δ	1073(e3)
003-005	CCA	V_B1-12_-D-J-V_B1-13_-δ	562(e2)
3.007		V-J-m-δ	1(e1)
3.169		V-(D)-J-δ	1(e1)
004-006	AGTG	V-(D)-δ	611(e2)
004-078	C	V(before RSS)-δ	236(e1)
006-049/55		δ only ?	337(e1)
006.078/82		δ only ?	623(e2)
006-106	CAG	V-(D?)-J-δ	begin(e1)
007-025	AGTGANGACACAG	V(before RSS)-δ	506(e2)
007-171	CATCAG	D-genomic-D-J-δ	647(e2)
9.03		V-?-δ	235(e1)
009-052	CCAC	V-(D?)-J-δ	294(e1)
11.169	CTG	V-(D)-J-δ	1089(e3)
12.059	GAC	V(before RSS)-δ	242(e1)
017-108	ACACA	V(before RSS)-δ	689(e2)
017-121	CAGAGG	V(before RSS)-δ	253(e1)

^a ^Joining position in exon1, 2, or 3.

^b ^Joining does not involve RSS but initiates earlier.

In up to 20% of the rearrangements the variable sequence was identified as from one locus and the constant sequence from the other locus. For example, Cμa was found rearranged with members of family 1 and 8 from the *IGH-B*, and Cμb was found rearranged with members of family 1 and 6 from the *IGH-A*. However, twenty analyzed EST sequences in our EST database [42] that contained a Cμ sequence, contained a VH sequence from the same locus (data not shown). The locus origin of the JH sequences in the rearrangements could not be unambiguously established. Nevertheless, in a number of these rearrangements, the point of crossover appears to be located in the amplified part of the Cμ sequence based on the five single nucleotide differences between the two loci.

### Expression of the Atlantic salmon Ig genes

**Figure 6:**
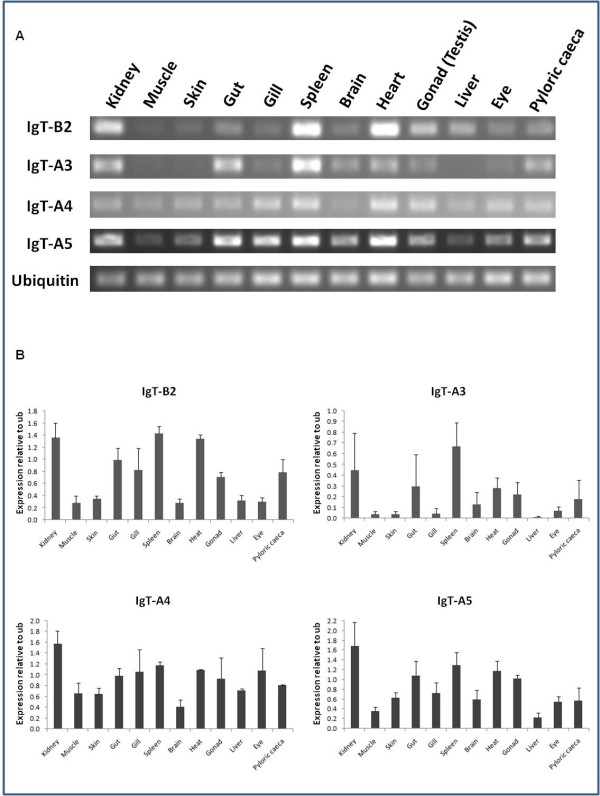
**Detection of Ig genes in various Atlantic salmon tissues by RT-PCR analysis**. (A) Agarose gel electrophoresis with PCR products of the Ig genes. Ubiquitin is an internal control. (B) The level of expression is calculated relative to the ubiquitin (ub) expression level. Data are expressed as mean ± SE of three fish.

The tissue distribution for four different forms of IgTs, IgT-B2, IgT-A3, IgT-A4 and IgT-A5, was examined by RT-PCR (Figure 6). Figure 6 represents the results from analysis of 12 tissues from 3 different adult individuals. The IgT genes were expressed at high levels in the kidney and spleen. It has been reported that other teleost *IgM *and *IgD *genes were also primarily expressed in kidney and spleen [16,18,22]. The kidney and spleen are the major lymphoid organs of teleosts [43,44]. The teleost anterior kidney is a main site for B lymphogenesis, while the teleost posterior kidney provides an environment capable of inducing B cell activation and differentiation into plasma cells [45]. The teleost spleen functions as a major secondary immune organ, as in mammalian species. Mature B cells are abundant at this site, and Ig-secreting cells have been detected from splenic B cells [46]. Interestingly, the *IgT *genes were also highly expressed in the mucosal tissues, such as the gut or gills (Figure 6). In other tissues, different expression patterns were observed among the different Cτ genes (Figure 6). In particular, the expression pattern of IgT-A3 was quite different from the other three IgTs (Figure 6). Interestingly, the constant region of the IgT-A3 (Cτ_A_-3) has a frameshift, and does not have any D segments. In addition, we could not find the IgT-A3 in VH cDNA clones. Therefore, the functionality of IgT-A3 must be questioned. It should also be noted that the expression of IgT-A4 was not detected in one fish. The expression of IgT-B2, IgT-A4 and IgT-A5 was highly expressed in the heart. Hansen et al. (2005) have suggested that the expression of Ig genes in the heart is most likely due to circulating B cells, because salmonid blood is a rich source of leukocytes [22]. In the present study, fish were bled before isolation of tissues but were not exsanguinated. It is therefore assumed that blood remained in the heart.

**Figure 7:**
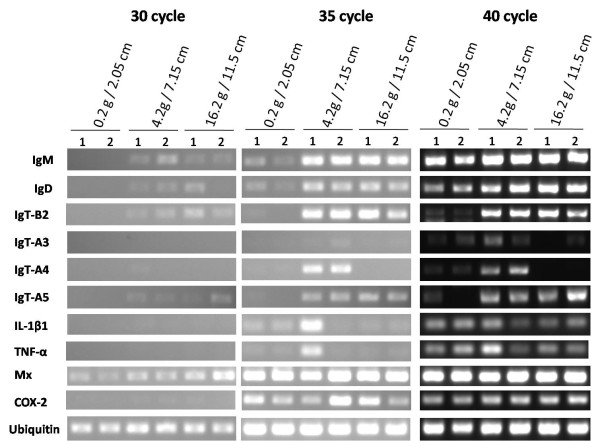
**Expression of Ig genes during three early developmental stages**. PCR amplifications of each primer set were performed for 30, 35 and 40 cycles. Two individuals were studied in each stage. Four immune related genes, *il-1β1 *(IL-1β1), *tnf-α *(TNF-α), *mx *(Mx) and *cox-2 *(COX-2), were also examined. Ubiquitin is an internal control.

We also examined the expression of Ig genes during three early developmental stages of Atlantic salmon by RT-PCR (Figure 7). IgM and IgD were weakly detected in the earliest stage (0.2 g/2.05 cm), and the expression of both genes increased at later stages of development. Similarly, the expression of both IgT-B2 and IgT-A5 was increased in these stages; however, the expression of these genes was negative or very weak in the earliest stage. Interestingly, the expression of IgT-A4 was not detected in the 16.2 g/11.5 cm fish, and at especially high levels in the 4.2 g/7.15 cm fish. Similarly, IgT-A3 was highly expressed in the 4.2 g/7.15 cm fish, and very weak expression of the gene was observed in only one individual of the 16.2 g/11.5 cm fish.

Four immune related genes, *il-1β1 *(IL-1β1), *tnf-α *(TNF-α), *mx *(Mx) and *cox-2 *(COX-2), were also examined (Figure 7). IL-1β, TNF-α and COX-2 are key mediators of the inflammatory response [47], while Mx proteins are members of the type I interferon (IFN)-inducible genes, and play a role in anti-viral defenses in teleosts [48]. The expression of IL-1β1 and TNF-α was quite similar with strong expression observed in only one individual of the 4.2 g/7.15 cm fish. Only very weak expression of these genes was observed in other fishes at different stages. The expression of Mx increased during the three developmental stages examined, while the expression of COX-2 was quite variable among individual fishes and development stage.

## Discussion

### Structure of the duplicated *IgH *loci, *IGH-A *and *-B*

**Figure 8:**
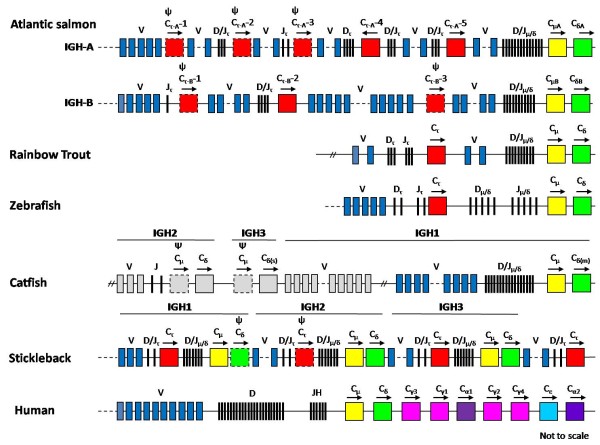
**Schematic structures of *IgH *loci of the Atlantic salmon and other vertebrates (not to scale)**. The Atlantic salmon duplicated *IgH *loci, *IGH-A *(670 kb) and *IGH-B *(710 kb), were completely sequenced in this study. The diagram of the *IgH *locus of zebrafish (175 kb), stickleback (175 kb) and human (1, 250 kb) are modified from references, [21,28,29] and [14], respectively. To date, only 3' end regions of approximately 100 kb in the rainbow trout [22] and 260 kb in the catfish [25]*IgH *locus have been sequenced. In the catfish locus, linkage among *IGH1*, *IGH2 *and *IGH3 *was established by restriction mapping and Southern blot analyses [26,82]. The complete sequences of the catfish *IGH2*, *IGH3 *and the upstream of *IGH1 *VH region have not yet been reported. Therefore, these regions are shown in gray boxes. The continuous regions are indicated by dotted lines, while the gap regions are indicated by double slashes (//). Dashed-line boxes indicate the CH pseudogenes (ψ). Transcription directions are shown by arrowhead.

Mammalian *IgH *loci do not have any CH genes located upstream of Cμ genes. Recently, however, a novel CH gene located upstream of Cμ and Cδ genes has been found in the *IgH *locus of zebrafish, rainbow trout and fugu [21-23]. In the stickleback *IgH *locus, a cluster of Cτ-Cμ-Cδ was found duplicated three times in tandem, with an additional Cτ gene in the 3' end of the locus [28,29]. Similarly, the catfish *IgH *locus contains three linked pairs of Cμ and Cδ genes, but a CH region upstream of Cμ and Cδ genes similar to IgT has not been found in that locus [25,26] (Figure 8). In this study we confirmed that two duplicated *IgH *loci can functionally coexist and further found that several novel CH (Cτ) genes exist between the VH and Cμ region of the duplicated Atlantic salmon *IgH *loci, five in *IGH-A *and three in *IGH-B*. Therefore, the Atlantic salmon duplicated *IgH *loci are the only *IgH *loci so far known, in which multiple Cτ genes are spread out over the region upstream of Cμ. Of these Cτ genes, two Cτ genes in *IGH-A *(Cτ_A_-4 and Cτ_A_-5) and one Cτ gene in *IGH-B *(Cτ_B_-2) are functional (Figure 8). These three genes were recovered in cDNA clones associated with VH, D and JH sequences. Interestingly, the Cτ_A_-4 gene is in the inverse orientation as has also been observed with the Cα genes in duck and chicken [49,50]. In addition, the inverted Cτ_A_-4 gene is found associated with VH and D sequences both 5' and 3' of the constant sequence, and the Cτ_A_-4 gene was the most frequently used Cτ gene in our present analysis of VH rearranged cDNAs. Thus, the Atlantic salmon expresses seven kinds of CH genes, three functional Cτ genes (Cτ_B_-2, Cτ_A_-4 and Cτ_A_-5), in addition to the two previously known Cμ genes (Cμ_A _and Cμ_B_) and Cδ genes (Cδ_A _and Cδ_B_)[17,31] (Figure 2 and Figure 8).

In the zebrafish and fugu *IgH *loci, VH genes are located upstream of the Dζ1 (zebrafish) or DH1 (fugu) segment [21,23]. However, both *IgH *loci in Atlantic salmon (Figure 1 and Figure 8) and the rainbow trout *IgH *locus [22] also have VH genes between the Cτ and Cμ genes, suggesting that these additional VH regions arose in the family Salmonidae. Interestingly, the Atlantic salmon VH region that contains the VH sequences coincides with the region that contains a large number of "Nhe I" elements, piggyBAC-like sequences (Figure 1) that are also highly concentrated in the V region of the Atlantic salmon *TRA/TRD *locus [37]. We postulate that these elements are involved in the generation and diversification of the large number of V segments of the Atlantic salmon *IgH *loci and *TRA/TRB *locus.

The two Atlantic salmon *IgH *loci contain over 300 VH genes, 99 VH sequences in *IGH-A *and 103 VH sequences in *IGH-B *were characterized in this study. These Atlantic salmon VH genes could be grouped into 18 families, based on >75% nucleotide identity (Figure 3). This is the largest number of VH families currently defined in any vertebrate. However, it includes a high number of pseudogenes (>68%). The proportion of pseudogenes is quite different among vertebrate species. The mouse locus contains 56% (110/195) functional genes [51], while human have 36% (44/123) [52]. In teleosts, the zebrafish locus contains 47 VH genes, of which 36 are presumed functional (77%) [21]. In catfish, there are 165 - 200 VH segments as estimated by Southern blotting, and the analysis of 10% of the germline VH genes suggested that approximately 50% are pseudogenes [53]. Although a large number of VH genes in the Atlantic salmon are pseudogenes, some of these pseudo VH genes were expressed. It is unclear whether these pseudo VH genes may play a functional role.

### Structural differences between *IGH-A *and *-B*

We found some structural differences between the two parallel *IgH *isoloci in Atlantic salmon. The orientation of the VH sequences indicates some rearrangement within the loci and an approximately 200 kb inversion event is evident in the *IGH-A *locus when comparing the *IGH-A *and *IGH-B *sequence (Figure 1 and Additional file 4). This inversion event, which ranges from approximately position 480 kb to 680 kb (in Figure 1) explains the inverse orientation of the Cτ_A_-4 sequence in *IGH-A*. In addition, three VH families, families 13, 14 and 18 were found only in *IGH-A *(Figure 3). An additional group of three D segments associated with the Cμ gene exists in the *IGH-A *locus (see Additional file 9). The amino acid sequence of Cμ_B _has an extra cysteine residue near the C-terminal end as described previously by Hordvik et al. (2002) [35] (see Additional file 5). This additional cysteine in Cμ_B _is absent in Cμ of rainbow trout [35]. Moreover, the amino acid sequence identities between the rainbow trout Cτ and the Atlantic salmon Cτ_A_s (Cτ_A_-4 and Cτ_A_-5) is higher (80 - 82%) than the similarity between the rainbow trout Cτ and the Atlantic salmon Cτ_B _(Cτ_B_-2) (75%). In addition, phylogenetic analysis indicates that Cτ_A_s are more closely related to the rainbow trout Cτ than to Cτ_B _(see Additional file 6). It has been reported that both duplicated *IgD *genes in Atlantic salmon have a tandem duplication of Cδ_2_-Cδ_3_-Cδ_4 _[17,38]. While our present study shows that these three exons (Cδ_2_-Cδ_3_-Cδ_4_) are indeed repeated three times in *IGH-A*, they are repeated four times in *IGH-B *(Figure 2). These observations indicate that both *IGH-A *and *-B *loci have evolved independently in the short time after the recent genome duplication. Thus, the existence of two parallel *IgH *isoloci in Atlantic salmon has contributed to the extensive diversity of the antibody repertoire.

### Atypical VH -D-JH -Cδ rearrangements in Atlantic salmon

Analysis of the RT-PCR products amplified using primers specific for Cδ and for VH genes showed that there were a large number of different (unexpected) band sizes and some multiple bands in PCR product from one set of primers (data not shown), indicating that various types of VH-D-JH-Cδ rearrangements exist in Atlantic salmon. The teleost *IgD *gene is expressed as a chimeric transcript that includes the first exon of *IgM *gene (Cμ_1_), because the teleost first exon of *IgD *(Cδ_1_) does not contain an appropriate cysteine expected to form the disulfide bond with the L chain [16-20]. Unexpectedly, analysis of the Atlantic salmon *IgD *VH cDNA clones revealed that approximately 10% of the *IgD *transcripts do not include the Cμ_1 _sequence, showing that the Atlantic salmon *IgD *can be expressed as both a chimera and without the inclusion of the Cμ_1 _sequence. Recently, it has been reported that either of the Cμ_1 _and Cδ_1 _exons could be observed in expressed porcine *IgD *cDNA sequences (VDJ-Cμ_1_-hinge region (H)-Cδ_2_-Cδ_3 _or VDJ-C δ_1_-H-Cδ_2_-C δ_3_) [54]. The porcine genomic Cδ_1 _exon is highly similar to the Cμ_1 _exon with only 4 nucleotides difference. Both the Cδ_1 _and Cμ_1 _exons contain three cysteines, only one of which becomes part of the *IgD *transcript and interacts with the L chain. In contrast, the amino acid sequences of the Atlantic salmon Cδ_1 _exons are quite different from that of the Cμ_1 _exons. In addition, the Cδ_1 _exon lacks the cysteine, similar to other teleosts [17]. Therefore, the functionality of these non-chimeric *IgD *transcripts is questionable. In fact, these non-chimeric *IgD *transcripts generally do not use the normal splice and recombination signals. In many of these non-chimeric *IgD *transcripts, joining takes place from somewhere inside or just after the VH sequence to various distributed sites inside exon 1, 2, or 3 of the Cδ sequence. In many of these instances, a short repeat sequence is present at the two joined ends (Table 2). We cannot at this time exclude the possibility of PCR artifacts contribute to these atypical VH-D-JH-Cδ sequences. However, because they are only observed with Cδ and not with Cτ and Cμ, we assume that it is not very likely. It will be of interest, in future studies, to discover the functions of these unusual *IgD *transcripts.

### Ig genes in Atlantic salmon

Teleost IgM molecules exist in both secreted and membrane-bound forms. The membrane forms of teleost IgM splice the transmembrane (TM1) exon directly to the Cμ_3 _exon splice site because of lack of a cryptic splice site within Cμ_4 _exon [55]. The Atlantic salmon and other teleost IgD have been identified as membrane *IgD *transcripts only [16,18,19,38]. In contrast, the catfish *IgH *locus encodes both membrane and secreted Cδ genes, and a secreted IgD molecule is identified in the serum of catfish [56]. However, the catfish secreted IgD molecule is encoded by a pseudo *IGHM-IGHD *locus and may not contain a functional VH region [26]. We found cDNAs encoding secreted and membrane-bound forms of the three functional IgTs in the Atlantic salmon EST database [42], indicating that these three IgTs exist in both the secreted and membrane-bound forms as has also been observed with the IgTs of zebrafish, rainbow trout and fugu [21-23]. Unlike teleost membrane-bound IgM, the membrane-bound forms of these three *IgT*s transcripts include Cτ_4 _exons as found in the IgTs of zebrafish and rainbow trout [21,22].

The novel Ig class (IgT), found in zebrafish, rainbow trout and fugu, possesses its own complement of D and JH segments [21-23]. This organization resembles that seen in the mouse *TRA/TRD *locus [21]. Similarly, each of the three functional Atlantic salmon IgTs has its own complement of D and JH segments (Figure 1 and Figure 8). In mammals, antigen-reactive B cells make antibodies of a single type as according to the "one cell-one antibody" rule [57]. If this rule applies to Atlantic salmon, a single B cell should only express one kind of Ig class from the three different IgTs or IgM, because these Igs have different D and JH segments. Li et al. (2006) found that the gene encoding the rainbow trout IgT was expressed only in IgM^- ^peripheral blood leukocytes (PBLs), indicating that those IgT^+^IgM^- ^cells represent a unique subset of lymphocytes [58]. Therefore, the Atlantic salmon may possess three different IgT^+^IgM^- ^B cell and IgT^-^IgM^+ ^B cell populations. If so, the mechanism of expression of the Atlantic salmon IgTs differs from the mammalian "class switch recombination" mechanism. However, it is not known whether or not the Atlantic salmon B cells only express single IgT or IgM. Further study on the Atlantic salmon B cells will provide new insights into the evolution of B cells in vertebrates.

### Expression of the three novel IgT isotypes in Atlantic salmon

The expression of the four innate-immune related genes was variable among individual fishes and development stages, while the expression of Ig genes were quite similar among individual fishes and developmental stages (Figure 7). The expression of the IgM, IgD and four innate immune-related genes were detected in the earliest stage (0.2 g/2.05 cm fish). On the other hand, the expression of the three IgTs was negative or very weak in the earliest stage (Figure 7), suggesting that the Atlantic salmon IgTs are involved in the more mature developmental stage than the IgM and IgD.

As mentioned above, three intact Cτ genes (Cτ_A_-4, Cτ_A_-5 and Cτ_B_-2,) were found in Atlantic salmon. The Cμ and Cδ genes, respectively, show a high degree of amino acid identities between *IGH-A *and *IGH-B *(96% ~) [17,31]. In contrast, the three intact Cτ genes (Cτ_B_-2, Cτ_A_-4 and Cτ_A_-5) exhibit significant sequence divergence at the amino acid level not only between loci but also within a locus (*IGH-A*) (see Additional file 5). The per cent amino acid identities between the Cτ_A _(Cτ_A_-4 and Cτ_A_-5) and Cτ_B _(Cτ_B_-2) sequences is 75-76, and between Cτ_A_-4 and Cτ_A_-5 is 87. RT-PCR analyses revealed different tissue distribution patterns among these three IgTs (Figure 6). In addition, the expression pattern of IgT-A4 was quite different from the other two IgTs during the three early developmental stages tested. The expression of IgT-A4 was not detected in the 16.2 g/11.5 cm fish (Figure 7). The high degree of amino acid diversity and the differential expression patterns among three Cτ genes suggest that the three different IgTs may have different functions.

Interestingly, in our present RT-PCR analysis, the IgTs, especially IgT-A5, were highly expressed in the mucosal tissues, including gut or gills (Figure 6). The fugu IgT mRNA was also strongly expressed in goblet cells of the intestine and gill epithelium [23]. In mammals, the mucosal surfaces constitute the first defensive line against invading microbial pathogens. The specific immunological defense at this site is primarily mediated by IgA antibodies [59,60]. Although the structure of the *Xenopus laevis *IgX is quite different from the mammalian IgA, the IgX is considered an analog of IgA because its association with the mucosae of the intestine resembles that of IgA [61]. In teleosts, no typical mucosal Ig class such as the IgA and IgX has been identified, but small amounts of IgM is present in gut mucus of several teleosts [62-64]. However, Hatten et al. (2001) have reported that IgM is not present in gut mucus of Atlantic salmon, and the gut mucus contains a large amount of proteolytic enzymes able to degrade serum IgM [65]. Their results suggested that antibodies related to the gut of the Atlantic salmon should be of another, yet unidentified, Ig class. Because the three new IgTs identified in our present study were found to be highly expressed in mucosal tissues, these IgTs might form the mucosal Ig class in Atlantic salmon.

## Conclusions

The present study shows that the genomic organization of the duplicated *IgH *loci in Atlantic salmon differs from that in other teleosts. The loci possess multiple Cτ genes upstream of the Cμ region, with three of the Cτ genes being functional. Moreover, the duplicated loci possess over 300 VH segments which could be classified into 18 families. This is the largest number of VH families currently defined in any vertebrate. Our results indicate that the duplication of the *IgH *loci in Atlantic salmon contributes heavily to the increases in diversity of the antibody repertoire, as compared with the single *IgH *locus in other vertebrates. Previous studies of the Atlantic salmon *TRA/TRD *and *TRG *loci revealed that Atlantic salmon clearly has one of the largest TCR repertoires known for any vertebrate [66,67]. Much more comprehensive analyses of Ig and TCR repertoires in Atlantic salmon can use the method of Warren et al. (2009) [68]. Atlantic salmon have both freshwater and saltwater phases in their life cycles. Therefore, Atlantic salmon is exposed to a wider variety of pathogens from these two different environments. Thus, the large diversity of antigen receptors in Atlantic salmon may have evolved to protect against such a wide variety of pathogens. Further study on the biological significance of the Igs and TCRs will provide unique insight into the evolution of the adaptive immune system in vertebrates.

## Methods

### Sequencing of Atlantic salmon *IGH-A *and *-B *loci

The Atlantic salmon *IGH-A *and *-B *loci were isolated and sequenced as previously described [66,67]. An Atlantic salmon BAC library (CHORI-214), constructed from a Norwegian aquaculture male strain, was obtained from BACPAC Resources, Children's Hospital Oakland Research Institute (CHORI) [69]. Six BAC library filters were hybridized with three (Cμ, Cδ and Cτ) 70-mer oligo probes (Integrated DNA Technologies) that were 5`-end-labeled with ^32^P-ATP using T4 polynucleotide kinase (Invitrogen). The labeled probes were added to BAC filters that had been pre-hybridized at 65°C for 4 h (5 × SSC, 5 × Denhardt's, 0.1% SDS). The hybridization was carried out overnight at 65°C. Three washes were performed, each for 30 min at 50°C; the first consisting of 2 × SSC and 0.1% SDS, and the second and third each consisting of 1 × SSC and 0.1% SDS. Filters were visualized using BioMax film (Kodak). BAC clones were chosen based on the physical BAC fingerprint map for Atlantic salmon [70] that is publicly available on the internet Contig Explorer (iCE) version 3.5 [71]. The BAC end sequence information, that is available in ASalBase [72], was also used for selection of the BAC clones. BAC shotgun libraries were constructed and sequenced on an ABI 3730 DNA sequencer, each of which was assembled using PHRED and PHRAP [73,74] and Consed [75].

The Dotter program [76] was used extensively to identify sequence elements. Sequence alignments were performed with ClustalW [77] and phylogenetic trees generated with MEGA3.1 [78] using the Unweighted Pair Group Method with Arithmetic Mean (UPGMA), pairwise deletion, and a p-distance model. Gene families were defined at 75% identity, as per the World Health Organization-International Union of Immunological Societies (WHO-IUIS) Nomenclature Subcommittee guidelines [79]. Genes flanking the loci were identified for *IGH-B *with the Digit Web Server [80].

The sequence of the *IGH-A *and *IGH-B *loci were deposited in Genbank under accession numbers, *IGH-A *[GenBank:GU129139], *IGH-B *[GenBank:GU129140] and the other *IGH-A *allele [GenBank:GU321975 - GU321980]. The nucleotide sequences for the Cμ, Cδ and Cτ probes were based on EST sequence data and are provided in Additional file 13.

### Cloning and sequencing of VH cDNAs

Adult Atlantic salmon (Mowi stock) tissues were obtained from the Department of Fisheries and Oceans (Robert Devlin, WestVan Lab., West Vancouver, British Columbia). Adult fish were euthanized, followed by rapid dissection of tissues. Tissues were flash frozen in liquid nitrogen or dry ice and stored at -80°C until RNA extraction. Total RNA was extracted from the kidney and spleen of two healthy individuals using TRIzol reagent (Invitrogen). Purified total RNA (1.0 μg) was reverse transcribed with SuperScript™ II (Invitrogen) using oligo (dT)15 primer as described in the manufacture's protocol. The cDNAs were synthesized in 25 μl reactions incubated at 42°C for 90 min and the transcriptase heat-inactivated at 70°C for 30 min. Equal amounts of each cDNA were combined and the mixture used as PCR template.

One hundred seventy eight (178) forward primers were designed from VH sequences identified for one or several VH sequences per primer (generally as part of a family). The reverse primers were designed from the consensus sequence of Cμ, Cδ, and Cτ. For increased specificity, nested primers were also designed from the consensus sequence of *IGH-A *and *-B *locus for Cμ and Cδ genes, and from the four different forms of Cτ genes. These nested primers were located in the first exon of τ (Cτ_1_), and in the second exon of μ (Cμ_2_) and δ (Cδ_2_). The PCR primers used in this study are shown in Additional file 13. PCRs were performed using GoTaq DNA polymerase (Promega) with an initial denaturation of 2 min at 95°C and then 30 cycles at 30 s of denaturation at 95°C, 30 s of annealing at 55°C, and 1 min of extension at 72°C. PCR products were cloned into pCR2.1 (TA Cloning Kit, Invitrogen) according to the manufacturer's protocol. Twelve (12) clones from each positive PCR product were sequenced as described above. Sequences obtained from Cμ and Cτ genes were sequenced in single forward direction, while the longer sequences that include the Cδ gene were sequenced in both forward and reverse directions. We only analyzed the results where the two reads could form a contig with a 100% overlap. The cDNA sequences were BLAST searched against the VH, JH and CH gene sequences to identify their presence in the clones.

### RT-PCR analysis of Ig genes expression

Twelve different Atlantic salmon tissues from three different adult fish were provided by the Department of Fisheries and Oceans. Immature and juvenile stages of fish were provided by Marine Harvest Canada (Big Tree Creek Hatchery, Sayward, B.C.) Total RNA was extracted and reverse transcribed using the method described above for tissues (kidney and spleen) from two individuals for three different development stages, as well as tissues from three individual adult fish (kidney, muscle, skin, gut, gill, spleen, brain, heart, gonad, liver, eye and pyloric caeca). PCR primers were designed from the consensus sequence of *IGH-A *and *IGH-B *locus for Cμ and Cδ genes, and four different forms of Cτ genes. The primers correspond to sequences in the first and third exon for Cμ and Cτ genes, and in the fifth and seventh exon for Cδ gene. Ubiquitin was used as internal positive control. In addition, four immune related genes, interleukin (IL)-1β1, tumour necrosis factor (TNF)-α, Mx and cyclo-oxygenase (COX)-2, were also examined. The PCR primers used in this study are shown in Additional file 13. PCR was performed using GoTaq DNA polymerase (Promega) with an initial denaturation step of 2 min at 95°C and then 30 or 35 cycles as follows: 30 s of denaturation at 95°C, 30 s of annealing at 55°C and 1 min of extension at 72°C. The PCR products derived from each primer set were TA-cloned and confirmed by sequencing. The PCR products were electrophoresed on a 1.0% agarose gel. The intensity of the amplification bands was semi-quantitatively measured using ImageJ software [81], and divided by the intensity of the respective ubiquitin signals.

## Abbreviations

*IgH*: immunoglobulin heavy chain locus; *IgL*: Immunoglobulin light chain locus; *IGH-A *and *-B*: Atlantic salmon duplicated immunoglobulin heavy chain loci A and B; VH: Variable region of immunoglobulin heavy chains; D: Diversity region of immunoglobulin heavy chains; JH: Joining region of immunoglobulin heavy chains; CH: Constant region of immunoglobulin heavy chains; Cμ: μ chain constant region; Cδ: δ chain constant region; Cγ: γ chain constant region; Cα: α chain constant region; Cε: ε chain constant region; Cζ: ζ chain constant region; Cτ: τ chain constant region; *TRB/TRD*: T cell receptor (TCR) *α/δ *locus, *TRG*, TCR γ locus.

## Authors' contributions

MY performed the BAC library preparation, VH cDNA cloning, RT-PCR studies and drafted the manuscript. JdB performed the data analysis and drafted the manuscript. KRVS performed the VH cDNA cloning and RT-PCR studies. GAC, LM, AM, SS performed the BAC library preparation and DNA sequencing for the project. WSD contributed to the project planning and direction. BFK contributed to the planning, design, and direction of the project. All authors read and approved the final manuscript.

## Supplementary Material

Additional file 1 **Features of the *IGH-A***. Table listing genes and pseudogenes identified in the *IGH-A.* Click here for file

Additional file 2 **Features of the *IGH-B***. Table listing genes and pseudogenes identified in the *IGH-B.* Click here for file

Additional file 3 **The identified genes flanking the loci**. Table listing genes flanking the loci identified with the Digit Web Server (http://synthetic-biology.jp/sw/pic/en/crib151s2rib151s72i/). Click here for file

Additional file 4 **Dotter plot of locus A (*IGH-A*) versus locus B (*IGH-B*)**. This file contains a dotter plot of *IGH-A *versus *IGH-B*. Click here for file

Additional file 5 **Alignment of amino acid sequences encoded by (A) Cτ, (B) C μ and (C) Cδ**. This file contains multiple sequence alignments of amino acid sequences encoded by (A) Cτ, (B) C μ and (C) Cδ obtained from ClustalW. Identical residues are shown as dots (.) and gaps are shown as hyphens (-). Click here for file

Additional file 6 **Phylogenic relationships for the CH genes in various species**. Phylogenic tree showing the relationship of the CH genes amino acid sequences of CH2 and CH3 domains of α, human δ and γ; CH3 and CH4 of μ, ζ/τ, ε, and duck α; CH4 and CH5 of new antigen receptor (NAR); CH5 and CH6 of ω, NARC and teleost δ. The tree was constructed with the MEGA 4 package by neighbor-joining (NJ) method and bootstrap values for replicated 1,000 were represented by percentages on the edge of node. The bootstrap values greater than 50% are presented. The scale bar indicates the branch length. Genbank accession numbers are as follows: *α*: duck [GenBank:AAA68606], human [GenBank:AAC82528]. *δ*: Atlantic salmon *δ*_A _and *δ*_B _[GenBank:AF278717; AF141605], catfish [GenBank:T18537], fugu [GenBank:BAD34542], zebrafish [GenBank:CAI11477], *Xenopus *[GenBank:DQ350886], human [GenBank:AAA52771]. *ε*: human [GenBank:AAB59395], opossum (*Monodelphis domestica*) [GenBank:AAC79674]. *γ1*: human [GenBank:AAC82527]. *γ3*: mouse [GenBank:AAB59697]. *μ*: Atlantic salmon *μ*_*A *_and *μ*_*B*_, [GenBank:AAB24064; AAF69490], bowfin (*Amia calva*) [GenBank:ACU12456], carp [GenBank:AB004105], catfish [GenBank:M27230], gar (*Lepisosteus osseus*) [GenBank:U12455], ladyfish (*Elops saurus*) [GenBank:M26182] lungfish (*Protopterus aethiopicus*) [GenBank:AF437724] nurse shark (*Ginglymostoma cirratum*) [GenBank:M92851], rainbow trout [GenBank:X83372], skate (*Leucoraja erinacea*) [GenBank:M29679], sturgeon (*Acipenser baeri*) [GenBank:Y13253], zebrafish [GenBank:AY643753], *Xenopus *[GenBank:M20484], chicken [GenBank:X01613], mouse [GenBank:J00443], human [GenBank:X14940]. *υ*: *Xenopus *[GenBank:X15114]. *ω*: lungfish [GenBank:AF437727], sandbar shark (*Carcharhinus plumbeus*) [GenBank:CPU40560]. *NAR*: nurse shark [GenBank:GCU51450]. *NARC*: nurse shark [GenBank:GCU18701]. *ζ/τ*: grass carp (*Ctenopharyngodon idella*) [GenBank:DQ489733], rainbow trout *τ1 *and *τ2 *[GenBank:AAW66978] and [GenBank:AAW66981], perch (*Siniperca chuatsi*), [GenBank:DQ016660], zebrafish [GenBank:AY643752]. Click here for file

Additional file 7 **Genbank accession numbers for deduced amino acid sequences of CH and VH domains**. Table listing the accession numbers for deduced amino acid sequences of CH and VH domains. Click here for file

Additional file 8 **Alignment of D sequences**. This file contains a multiple sequence alignment of D sequences obtained from ClustalW. Click here for file

Additional file 9 **Phylogenic trees showing the relationship between the (A) D and (B) JH sequences**. This file contains phylogenic trees for (A) D and (B) JH genes. Click here for file

Additional file 10 **Alignment of JH sequences**. This file contains a multiple sequence alignment of JH sequences obtained from ClustalW. Click here for file

Additional file 11 **Alignment of amino acid VH sequences**. This file contains a multiple sequence alignment of amino acid VH sequences obtained from ClustalW. Identical residues are shown as dots (.) and gaps are shown as hyphens (-). Click here for file

Additional file 12 **Distribution of variable (V**_**H**_**) families in the two *IgH *loci**. Table showing the number of sequences identified per family. Click here for file

Additional file 13 **PCR primers and oligo probes**. Table listing the PCR primers and oligo probes used in this study. Click here for file
