# Supplementary material for: Evolution of duplicated IgH loci in Atlantic salmon, Salmo salar
Source: BMC Genomics. 2010 Sep 2;11:486. doi: 10.1186/1471-2164-11-486 (PMC2996982; doi:10.1186/1471-2164-11-486)
Supplement: Additional file 3 — The identified genes flanking the loci. Table listing genes flanking the loci identified with the Digit Web Server (http://synthetic-biology.jp/sw/pic/en/crib151s2rib151s72i/). [file 1471-2164-11-486-S3.PDF]

**Putative genes 5' of the *IGH-B***	
212 - 10397	similar to CD97 antigen
36976 - 50104	solute carrier family 27 (fatty acid transporter)
52491 - 58020	tripartite motif containing 39
60120 - 63898	tripartite motif containing 35 (hemopoietic lineage switching protein
79705 - 98491	GSPT1 (G1 to S phase transition 1 isoform 1)
102461 - 118312	pyridoxal-dependent decarboxylase domain containing protein 1
136207 - 146638	solute carrier family 25 (fatty acid transporter)
222380 - 224724	Yip1 family protein
228285 - 241294	coactivator-associated arginine methyltransferase 1
254819 - 259298	caseinolytic protease
265495 - 267983	aldehyde dehydrogenase
**Putative genes 3' of the *IGH-B***	
1007576 - 1102505	Transcriptional repressor protein
1041767 - 1105704	Calcium channel protein
