# Supplementary figures and images for: Evolution of duplicated IgH loci in Atlantic salmon, Salmo salar
Source: BMC Genomics. 2010 Sep 2;11:486. doi: 10.1186/1471-2164-11-486 (PMC2996982; doi:10.1186/1471-2164-11-486)

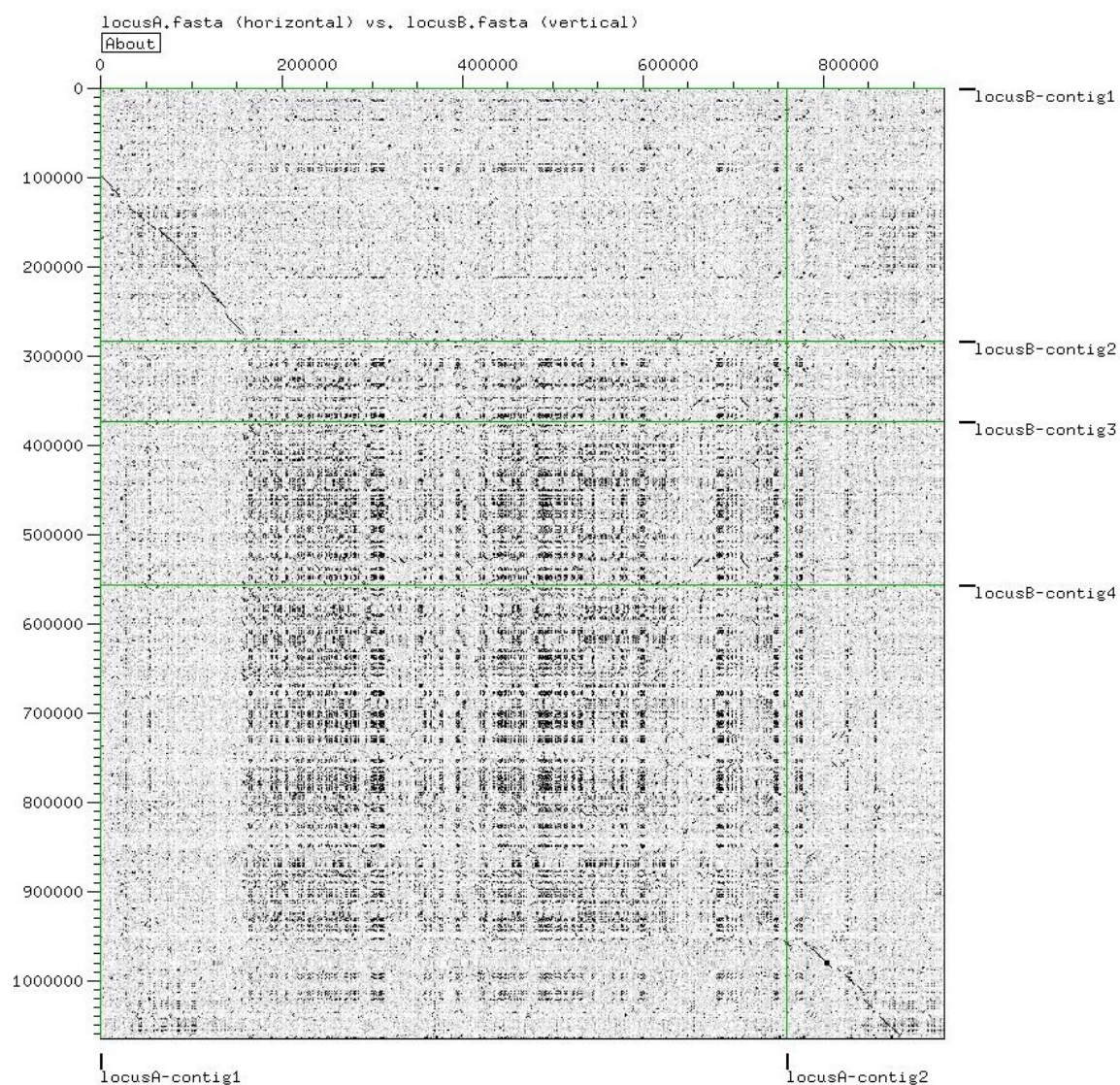

Supplement: Additional file 4 — Dotter plot of locus A (IGH-A) versus locus B (IGH-B). This file contains a dotter plot of IGH-A versus IGH-B. [file 1471-2164-11-486-S4.PDF]

**A**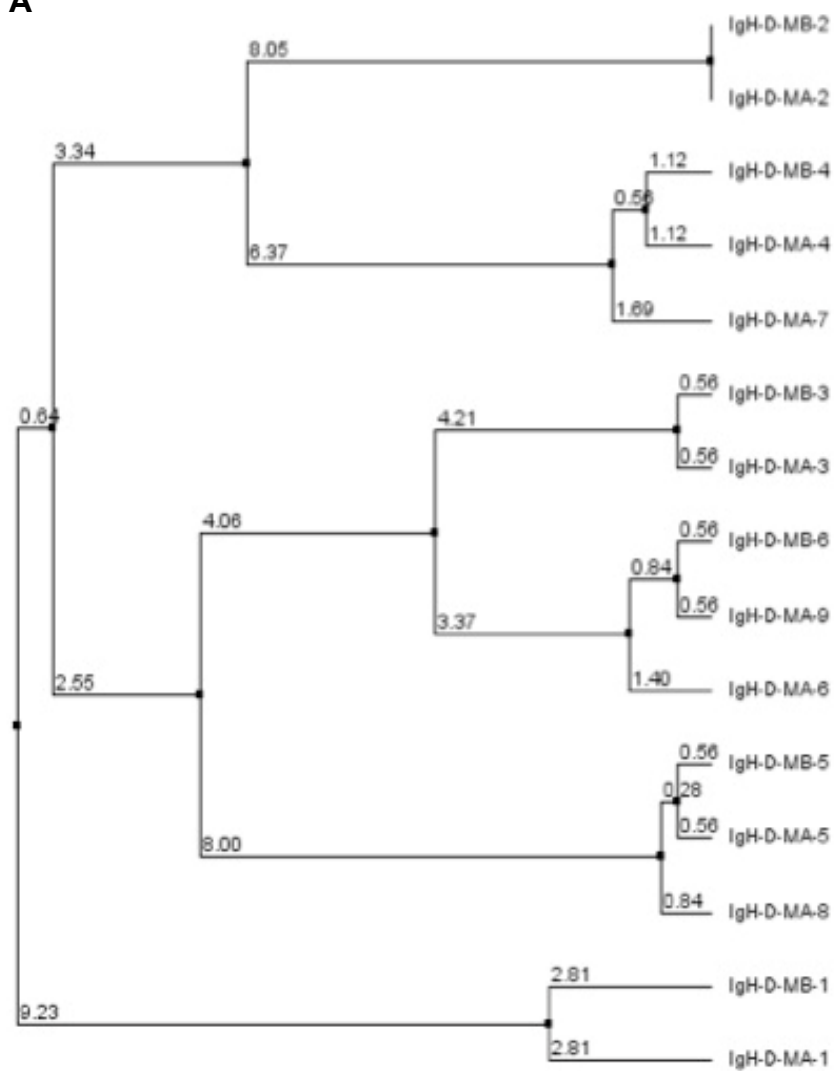**B**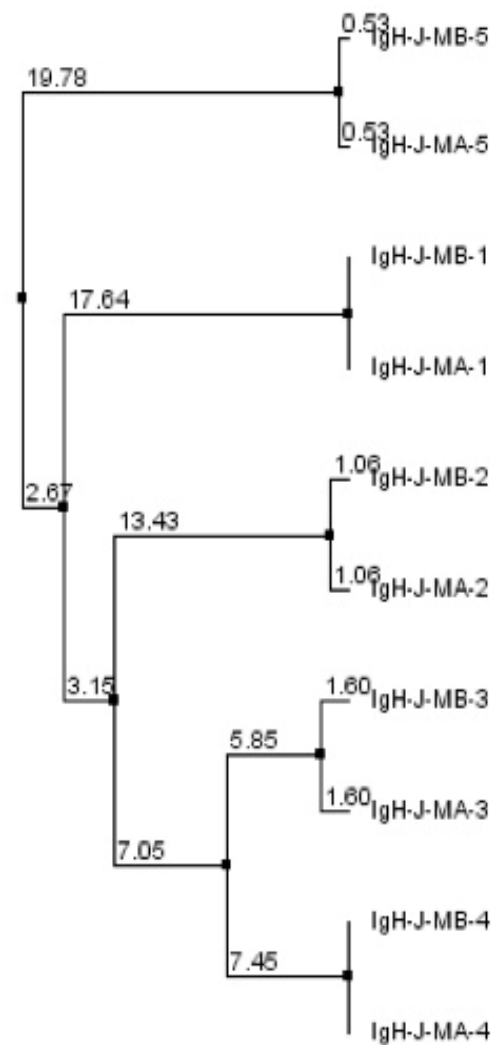

Supplement: Additional file 9 — Phylogenic trees showing the relationship between the (A) D and (B) JH sequences. This file contains phylogenic trees for (A) D and (B) JH genes. [file 1471-2164-11-486-S9.PDF]
