# Supplementary material for: Evolution of duplicated IgH loci in Atlantic salmon, Salmo salar
Source: BMC Genomics. 2010 Sep 2;11:486. doi: 10.1186/1471-2164-11-486 (PMC2996982; doi:10.1186/1471-2164-11-486)
Supplement: Additional file 5 — Alignment of amino acid sequences encoded by (A) Cτ, (B) C μ and (C) Cδ. This file contains multiple sequence alignments of amino acid sequences encoded by (A) Cτ, (B) C μ and (C) Cδ obtained from ClustalW. Identical residues are shown as dots (.) and gaps are shown as hyphens (-). [file 1471-2164-11-486-S5.PDF]

A. Cτ

```
                                      τ1
τA-4 AATTAPSSLF PLMNCGTPSN DIYSIGCVAT GFSPSSITFK WTDASESPLT DFVQYPSVQS  60
τA-5 .........L T......... ....L....K ......H... .....GKA.. ......A...  60
τB-2 ......PT.L T......... NV.....L.. ......L... .....R.T.. ......A...  60

                                                             τ2
τA-4 GGAYIGVSQV RVSKNDWEKS KSFRCSVEHP GGGKTAVIKK TVPKSPTVSL LSAPIGTTQY  120
τA-5 ..T.T....L ..A.SV..MT ...S...D.A .VV.....NR PISNP..... ..........  120
τB-2 ..T.T....L ..A.DV.DTA T..H...... ........N. P.S.P..... ..G.......  120

τA-4 LMCMIEDFTS NTVTVTWKKN DMEVEGQTPT LVKQPSGLYS GSSLLKVINT NWNNKVKYSC  180
τA-5 ...I....AP KK...S.... .KV...L... VGL.L..... .......N.. D.........  180
τB-2 ........AP .K........ ET....P... VGQ.....F. A......... D.........  180

                                            τ3
τA-4 VVQHQEQTIN KTISKTEPLT VTLNPPRVRE VFLDNQAVLE CVITGTDQDT VSGTTITWQV  240
τA-5 .......... .......... L.....S.KK ..I.....MD ...S...... ........H.  240
τB-2 .....G..TI .......... ......S.KK ..M......D ....A..... ....N.....  240

τA-4 NGEDKMDGID LKNIESKGNL NSRVSTLTIG QTEWTNVNKV QCSAMKSGED TPVIQDLSFT  300
τA-5 ..RKQT.H.. ..D....... .R........ .....K.... ......R... .......---  297
τB-2 .EQA.T.DV. ..P....... .........N .M........ .......... ...V.E....  300

                                            τ4
τA-4 KGSVAPSVSV HLLPEEDTKK EGEVTLVCLV VCPSLCDVYI MWQVD----- -SGQYQEGVT  354
τA-5 ---E...... .......... .......... .......... .....----- -.........  348
τB-2 ...Q...... ..V.K..P-L ......L... LS........ ..K.GKVGKV GEDN......  359

τA-4 SPPQKTQKAN YFVTSVFTTT KDKWERNLVF TCAVKHAGSD NNTALKERRV SKSMGNSCED  414
τA-5 ..L.....G. .S........ ..T..T.VL. ........L. .....MG.S. ...L......  408
τB-2 ........GS .L........ .NV.DTEVL. ....R.DSL. ....S..DS. ...K....Q.  419
                                                        Secretory tail
τA-4 K 415
τA-5 M 409
τB-2 M 420
```

τTM

```
τA-4 ANILLTEPEA GFALSCTDND EDEFSSLWST TSSFIILFLL SLTYSTVLSL VKMKQ 55
τA-5 E......... .......... ....G..... .......... .......... ..... 55
τB-2 E..P...... S......... .......... .......... .......... ..... 55
```

B. Cμ

```
                                      μ1
    <-----------------------------------------------------------------
μA  ASSTAPTLFP LAQCGSGTGD MMTLGCIATG FTPASLTFKW NEQGGNSLTD FVQYPAVQTS  60
μB  .......... .......... .V........ .......... .......... ..........  60

                                                                  μ2
    ------------------------------------------>  <--------------------
μA  GSYMGVSQLR VKRADWDSKI FECAVEHSAG SKTVPVKKQA EYLQHPSLYV MTPSKEEMAE 120
μB  .......... .......... .......... .....L...V .......... .......... 120

    -----------------------------------------------------------------
μA  NMTASFACFA NDFSPRTHTI KWMRMEQGIE KEVVSDFKSS CESEKKSDKT LYSTTSYLRV 180
μB  .K........ .......... ......K... .......... .......E.. .......... 180

                                                                  μ3
    --------------------------------------><-------------------------
μA  NESEWKSEEV AFTCVFENKA GNVRRTVGYT SSDAGPVHAH SVVIKITPPS LEDMLMNKKA 240
μB  .......... S.....K... .......... .......... ....N.I... .......... 240

    -----------------------------------------------------------------
μA  ELVCDVEELV PGFMSVKWEN DNGKTLTSRK GVTDRIAILD ITYEDWSNGT VFYCAVDHLE 300
μB  ......K... T......... .........M ....K..... .......... .......... 300

                                            μ4
    -------------------><---------------------------------------------
μA  NLGSLVKKPY KRETGGDPQR PSVFLLAPAE KTSDNTVTLT CYVKDFYPKE VLVAWLIDDE 360
μB  .......... .......... .......... .......... .......... .......... 360

    -----------------------------------------------------------------
μA  PVERTSSSAL YQFNTTSQIQ TGRTYSVYSQ LTFSNDLWKN KEVVYSCVVY HESMIKSTKI 420
μB  .......... .......... S......... .......... .......... .......... 420

    -------------------------------->
μA  LMRTIDRTSN QPYLVNLSLN VPQSCKAQ 448
μB  .......... ..N....... ...C.... 448
```

Secretory tail

μTM

```
μA  CLVLTDCPCS NTIETDRDSM GKTAFTFIIL FLITLLYGVG ATAIKVK 47
μB  .......... ..M....... .......... .......... ....... 47
```

C. Cδ

δ1

```
δ1A QRVIPPNITL YPLWEELEGG SKVGLLCILS EFYPDKLSVE WLLDDKTVTT YPVQRKLQSV 60
δ1B .......... .......... .......... G......... .......A.. S......... 60

δ1A EGEEKTFSLN SQLELDQSQW TQGSEVTCKA IHNAAQGPHP GTTVSRTISI CS 112
δ1B .......... .......G.. .......... T.......P. .......... .. 112
```

δ2

```
δ2-1A FPSSTPSLHL ETPRFRTVMT QTEVTATCVV HSAYDAKVSW HLDGKDPTSR TPVNQASSTT 60
δ2-2A .......... .......... .......... .......... L......... .......... 60
δ2-3A .......... .......... .......... .......... L......... .......... 60
δ2-1B -......I.. .......... .......... ........A. L......... .......... 59
δ2-2B .......... .....M.... .......... .......... L......... .......... 60
δ2-3B .......... .....M.... .......... .......... L......... .......... 60
δ2-4B .......I.. .......... .......... .....T.... L......... .......... 60

δ2-1A QSISSNLTLP SSQWKTLNTI TCRAEHRCFN PTQRTSNVN 99
δ2-2A .......... .......... .......... ......... 99
δ2-3A .......... .......... .......... ......... 99
δ2-1B .......... .......... .......... R.......K 98
δ2-2B .......... .......... .......... R.......K 99
δ2-3B .......... .......... .......... R.......K 99
δ2-4B .......... .......... .......... R.......K 99
```

δ3

```
δ3-1A TAVTSTPTVL IRRSLPDLLD GDSAVLECAI TQLSSSDLYV TFQANGVDFP EKQYVDLPAS 60
δ3-2A P......... .......... .......... .......... .......... .......... 60
δ3-3A P......... .......... .......... .......... .......... .......... 60
δ3-1B P..S...... .......... .......... .......I.. .......... .......... 60
δ3-2B P..S...... .......... .......... .......I.. .......... .......... 60
δ3-3B P..S...... .......... .......... .......... .......... .......... 60
δ3-4B P..S...... .......... .......... .......... .......... .......... 60

δ3-1A KDHHSLTRRF SIPTSHWKKE NTFTCKVNQG YSNSWVSNST GTLF 104
δ3-2A ..P....... .......... .......... .......... .... 104
δ3-3A ..P....... .......... .......... .......... .... 104
δ3-1B .......... .........D .......... .......... .--- 101
δ3-2B .......... .........D .......... .......... .--- 101
δ3-3B .......... .........D .......... .......... .... 104
δ3-4B .......... .........D .......... .......... .... 104
```

δ4

```
δ4-1A ELSMELLLVP NEEMSGSGTQ KLMCSGRGFN PQIKWLSG-- ------SKQR SAADNERRMR 52
δ4-2A .......... .......... .......... ........-- ------.... .......... 52
δ4-3A .......... .......... .......... .......VSA AAYEIR.... .......... 60
δ4-1B .P........ ...L...... .......... ........-- ------.N.. .......... 52
δ4-2B .P....F... ...L...... .......... ........-- ------.N.. .......... 52
δ4-3B .P........ ...L...... .......... ........-- ------.N.. .......... 52
δ4-4B .P........ ...L...... .......... .......V-- ---------- ...AY.I..G 48

δ4-1A EDGHVAVTSH ITVTQQEWNE GKDFICEVID KDLQKT---- VRKSTS--- 94
δ4-2A .......... .......... .......... ......---- ......LCT 97
δ4-3A .......... .........Q ....T..... .Y...----- --------- 95
δ4-1B .......... .......... .......... ......---- ......LCT 97
δ4-2B .......... .......... .......... ......---- ......LCT 97
δ4-3B .......... .......... .......... ......---- ......LCT 97
δ4-4B ..ER...... .........Q ..N.T..... .Y...EDLQK ....INICA 97
```

δ5

```
δ5A TPISSQKVAV YLQGPTLQEL RTDGQVPVTC LLVGPSLGDF SVSWKVDGFV ASQGGVTRAP 60
δ5B ........G. .......... ..G....... .......... .......... .......... 60

δ5A KDHSNGTQTE QIMFNVSARD WHAHKLVSCE VKHRCSSQAQ VEHITKCR 108
δ5B .......... .......... .......... .......... ........ 108
```

δ6

```
δ6A PKPPSIKIVR PSVSDLWGSN NATLLCLVSG FFPSDVIVNW EKAGSRLPFS RYSSIPSVLY 60
δ6B .......... ..D....... .......... .......... .......... .......F.. 60

δ6A AGSSTYSMNS RLIVPRSEWD HNSNYSCAVR HESSERPITS TIENVF 106
δ6B .......... .......... Q......... .........H ...... 106
```

δ7

```
δ7A SVTPSAPTAT LLQGPSELVC LVLGFSSSDI NITWLLDNVT ELWNNNTSTT YRAPGGKFGI 60
δ7B .......... .......... ......P... .......... .......... .......... 60

δ7A RSHLSLAHQD WTPGAVYTCR VTHTTQTLAL NISKP 95
δ7B .......... .......... .......... ..... 95
```

δ TM

```
δTM-A LLGVEGVFFD ENRSDPILAD TAEENWNMAC IFLVLFLISL LYSITVTLVK TK-- 52
δTM-B -......... .......... .......... .......... .......... DTSI 54
```
