# Supplementary material for: Evolution of duplicated IgH loci in Atlantic salmon, Salmo salar
Source: BMC Genomics. 2010 Sep 2;11:486. doi: 10.1186/1471-2164-11-486 (PMC2996982; doi:10.1186/1471-2164-11-486)
Supplement: Additional file 6 — Phylogenic relationships for the CH genes in various species. Phylogenic tree showing the relationship of the CH genes amino acid sequences of CH2 and CH3 domains of α, human δ and γ; CH3 and CH4 of μ, ζ/τ, ε, and duck α; CH4 and CH5 of new antigen receptor (NAR); CH5 and CH6 of ω, NARC and teleost δ. The tree was constructed with the MEGA 4 package by neighbor-joining (NJ) method and bootstrap values for replicated 1,000 were represented by percentages on the edge of node. The bootstrap values greater than 50% are presented. The scale bar indicates the branch length. Genbank accession numbers are as follows: α: duck [GenBank:AAA68606], human [GenBank:AAC82528]. δ: Atlantic salmon δA and δB [GenBank:AF278717; AF141605], catfish [GenBank:T18537], fugu [GenBank:BAD34542], zebrafish [GenBank:CAI11477], Xenopus [GenBank:DQ350886], human [GenBank:AAA52771]. ε: human [GenBank:AAB59395], opossum (Monodelphis domestica) [GenBank:AAC79674]. γ1: human [GenBank:AAC82527]. γ3: mouse [GenBank:AAB59697]. μ: Atlantic salmon μA and μB, [GenBank:AAB24064; AAF69490], bowfin (Amia calva) [GenBank:ACU12456], carp [GenBank:AB004105], catfish [GenBank:M27230], gar (Lepisosteus osseus) [GenBank:U12455], ladyfish (Elops saurus) [GenBank:M26182] lungfish (Protopterus aethiopicus) [GenBank:AF437724] nurse shark (Ginglymostoma cirratum) [GenBank:M92851], rainbow trout [GenBank:X83372], skate (Leucoraja erinacea) [GenBank:M29679], sturgeon (Acipenser baeri) [GenBank:Y13253], zebrafish [GenBank:AY643753], Xenopus [GenBank:M20484], chicken [GenBank:X01613], mouse [GenBank:J00443], human [GenBank:X14940]. υ: Xenopus [GenBank:X15114]. ω: lungfish [GenBank:AF437727], sandbar shark (Carcharhinus plumbeus) [GenBank:CPU40560]. NAR: nurse shark [GenBank:GCU51450]. NARC: nurse shark [GenBank:GCU18701]. ζ/τ: grass carp (Ctenopharyngodon idella) [GenBank:DQ489733], rainbow trout τ1 and τ2 [GenBank:AAW66978] and [GenBank:AAW66981], perch (Siniperca chuatsi), [GenBank:DQ016660], zebrafish [GenBank:AY643752] [file 1471-2164-11-486-S6.PPT]

## Slide 1
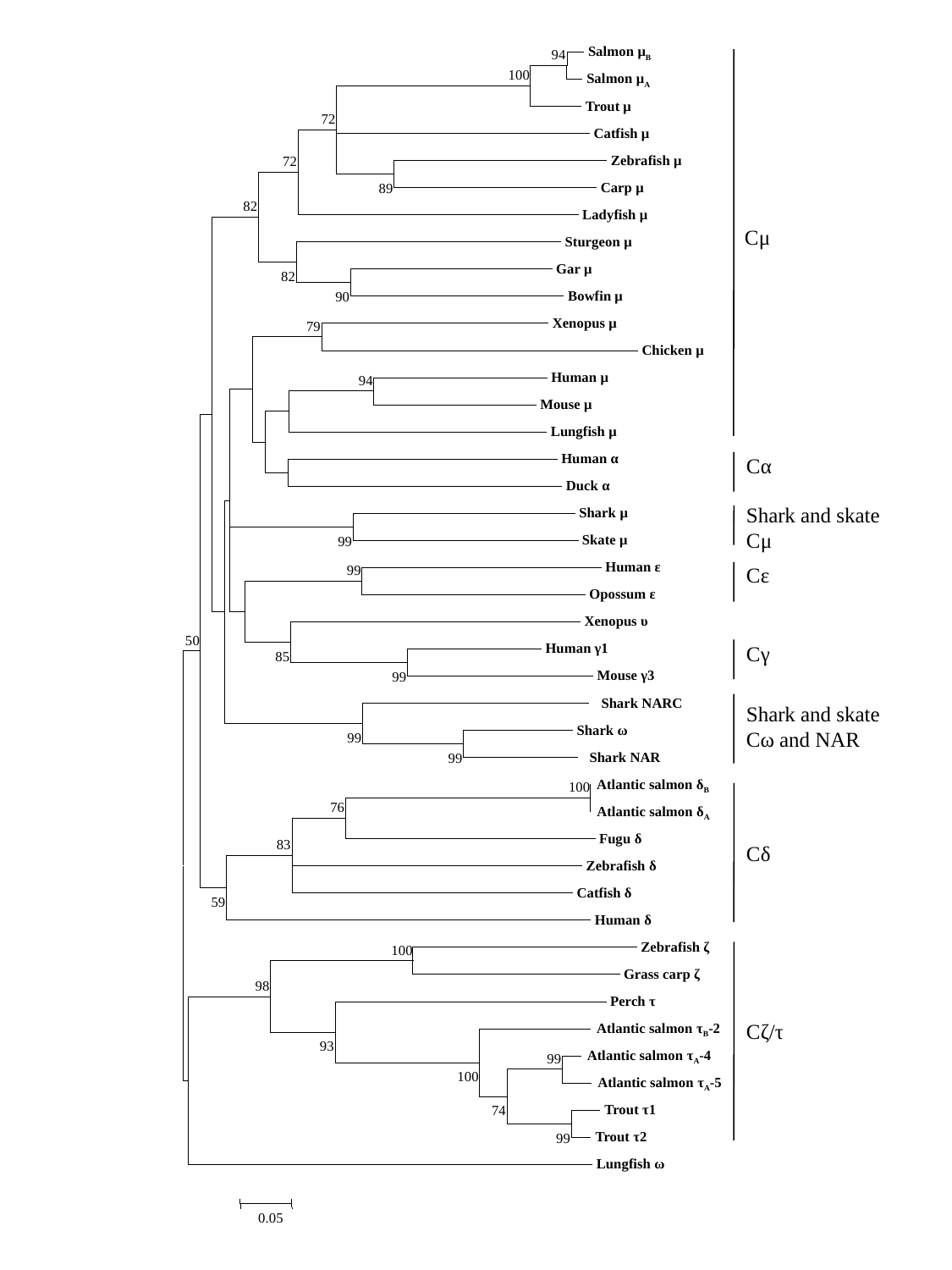

Salmon μB
94
100
 Salmon μA
 Trout μ
72
 Catfish μ
 Zebrafish μ
72
 Carp μ
89
82
 Ladyfish μ
 Sturgeon μ
 Gar μ
82
 Bowfin μ
90
 Xenopus μ
79
 Chicken μ
 Human μ
94
 Mouse μ
 Lungfish μ
 Human α
 Duck α
 Shark μ
 Skate μ
99
 Human ε
99
 Opossum ε
 Xenopus υ
50
 Human γ1
85
 Mouse γ3
99
 Shark NARC
 Shark ω
99
 Shark NAR
99
 Atlantic salmon δB
100
76
 Atlantic salmon δA
 Fugu δ
83
 Zebrafish δ
 Catfish δ
59
 Human δ
 Zebrafish ζ
100
 Grass carp ζ
98
 Perch τ
 Atlantic salmon τB-2
93
 Atlantic salmon τA-4
99
100
 Atlantic salmon τA-5
 Trout τ1
74
 Trout τ2
99
 Lungfish ω
0.05
Cμ
Cα
Shark and skate
Cμ
Cε
Cγ
Shark and skate
Cω and NAR
Cδ
Cζ/τ
