# Supplementary material for: Evolution of duplicated IgH loci in Atlantic salmon, Salmo salar
Source: BMC Genomics. 2010 Sep 2;11:486. doi: 10.1186/1471-2164-11-486 (PMC2996982; doi:10.1186/1471-2164-11-486)
Supplement: Additional file 7 — Genbank accession numbers for deduced amino acid sequences of CH and VH domains. Table listing the accession numbers for deduced amino acid sequences of CH and VH domains. [file 1471-2164-11-486-S7.PDF]

Genbank accession numbers for deduced amino acid sequences of CH and VH domains

Discription	Name	Accession no.	Discription	Name	Accession no.
CH region	CμA	ADD59861	VH region	IGHBV2-05	ADD59888
	CμB	ADD59895		IGHBV2-06	ADD59886
	CδA	ADD59862		IGHBV4-03	ADD59869
	CδB	ADD59896		IGHBV4-04	ADD59881
	CτB-2	ADD59873		IGHBV4-08	ADD59868
	CτA-4	ADD59858		IGHBV4-09	ADD59876
	CτA-5	ADD59859		IGHBV6-06	ADD59891
VH region	IGHVA1-05	ADD59857		IGHBV6-07	ADD59863
	IGHVA1-07	ADD59838		IGHBV6-08	ADD59871
	IGHVA1-08	ADD59843		IGHBV6-13	ADD59892
	IGHVA1-10	ADD59854		IGHBV6-14	ADD59867
	IGHAV1-14	ADD59847		IGHBV6-15	ADD59874
	IGHVA3-01	ADD59839		IGHBV8-02	ADD59866
	IGHVA4-08	ADD59844		IGHBV8-03	ADD59883
	IGHVA6-02	ADD59848		IGHBV9-01	ADD59884
	IGHVA6-04	ADD59851		IGHBV9-07 exon1	ADD59879
	IGHVA6-10	ADD59837		IGHBV10-04	ADD59880
	IGHVA7-02	ADD59845		IGHBV12-01	ADD59882
	IGHVA7-03	ADD59860		IGHBV15-03	ADD59865
	IGHVA8-01	ADD59842		IGHBV16-04	ADD59877
	IGHVA8-02	ADD59850		IGHBV16-07	ADD59885
	IGHVA8-03	ADD59852		IGHBV16-11	ADD59890
	IGHVA8-05	ADD59855		IGHBV16-13	ADD59875
	IGHVA8-07	ADD59841			
	IGHVA8-08	ADD59849			
	IGHVA8-11	ADD59840			
	IGHVA8-12	ADD59853			
	IGHVA8-13	ADD59856			
	IGHVA15-01	ADD59836			
	IGHVA17-01	ADD59846			
	IGHBV1-01	ADD59889			
	IGHBV1-02	ADD59870			
	IGHBV1-06	ADD59864			
	IGHBV1-07	ADD59878			
	IGHBV1-09	ADD59872			
	IGHBV1-13	ADD59893			
	IGHBV1-14	ADD59887			
	IGHBV2-01	ADD59894			
