# Supplementary material for: Evolution of duplicated IgH loci in Atlantic salmon, Salmo salar
Source: BMC Genomics. 2010 Sep 2;11:486. doi: 10.1186/1471-2164-11-486 (PMC2996982; doi:10.1186/1471-2164-11-486)
Supplement: Additional file 8 — Alignment of D sequences. This file contains a multiple sequence alignment of D sequences obtained from ClustalW. [file 1471-2164-11-486-S8.PDF]

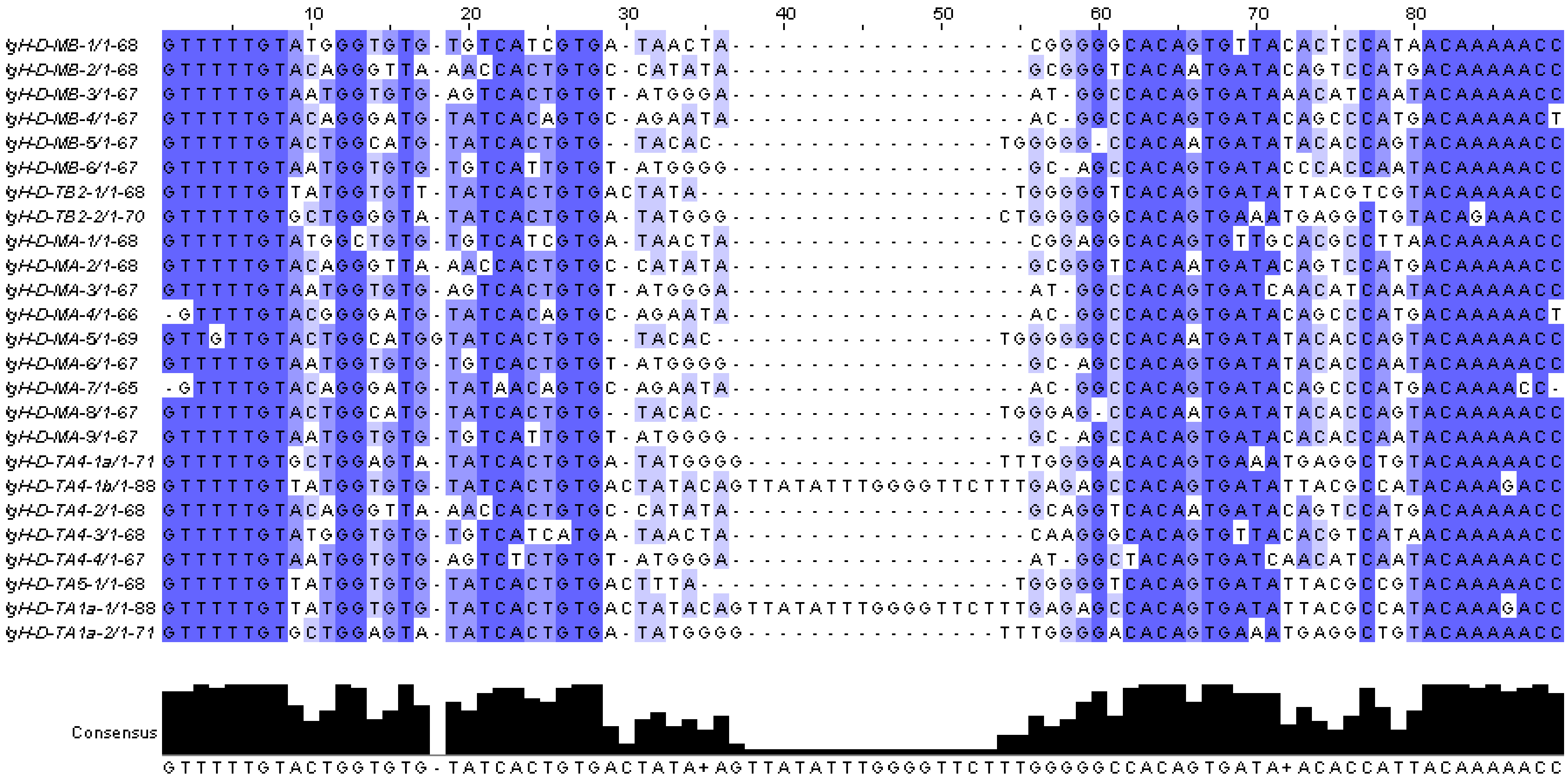

10
20
30
40
50
60
70
80
IgH-D-MB-1/1-68
IgH-D-MB-2/1-68
IgH-D-MB-3/1-67
IgH-D-MB-4/1-67
IgH-D-MB-5/1-67
IgH-D-MB-6/1-67
IgH-D-TB2-1/1-68
IgH-D-TB2-2/1-70
IgH-D-MA-1/1-68
IgH-D-MA-2/1-68
IgH-D-MA-3/1-67
IgH-D-MA-4/1-66
IgH-D-MA-5/1-69
IgH-D-MA-6/1-67
IgH-D-MA-7/1-65
IgH-D-MA-8/1-67
IgH-D-MA-9/1-67
IgH-D-TA4-1a/1-71
IgH-D-TA4-1b/1-88
IgH-D-TA4-2/1-68
IgH-D-TA4-3/1-68
IgH-D-TA4-4/1-67
IgH-D-TA5-1/1-68
IgH-D-TA1a-1/1-88
IgH-D-TA1a-2/1-71
Consensus
GTTTTTGTACTGGTGTG-TATCACTGTGACTATA+AGTTATATTTGGGGTTCTTTGGGGGCCACAGTGATA+ACACCATTACAAAAACC
