# Supplementary material for: Evolution of duplicated IgH loci in Atlantic salmon, Salmo salar
Source: BMC Genomics. 2010 Sep 2;11:486. doi: 10.1186/1471-2164-11-486 (PMC2996982; doi:10.1186/1471-2164-11-486)
Supplement: Additional file 10 — Alignment of JH sequences. This file contains a multiple sequence alignment of JH sequences obtained from ClustalW. [file 1471-2164-11-486-S10.PDF]

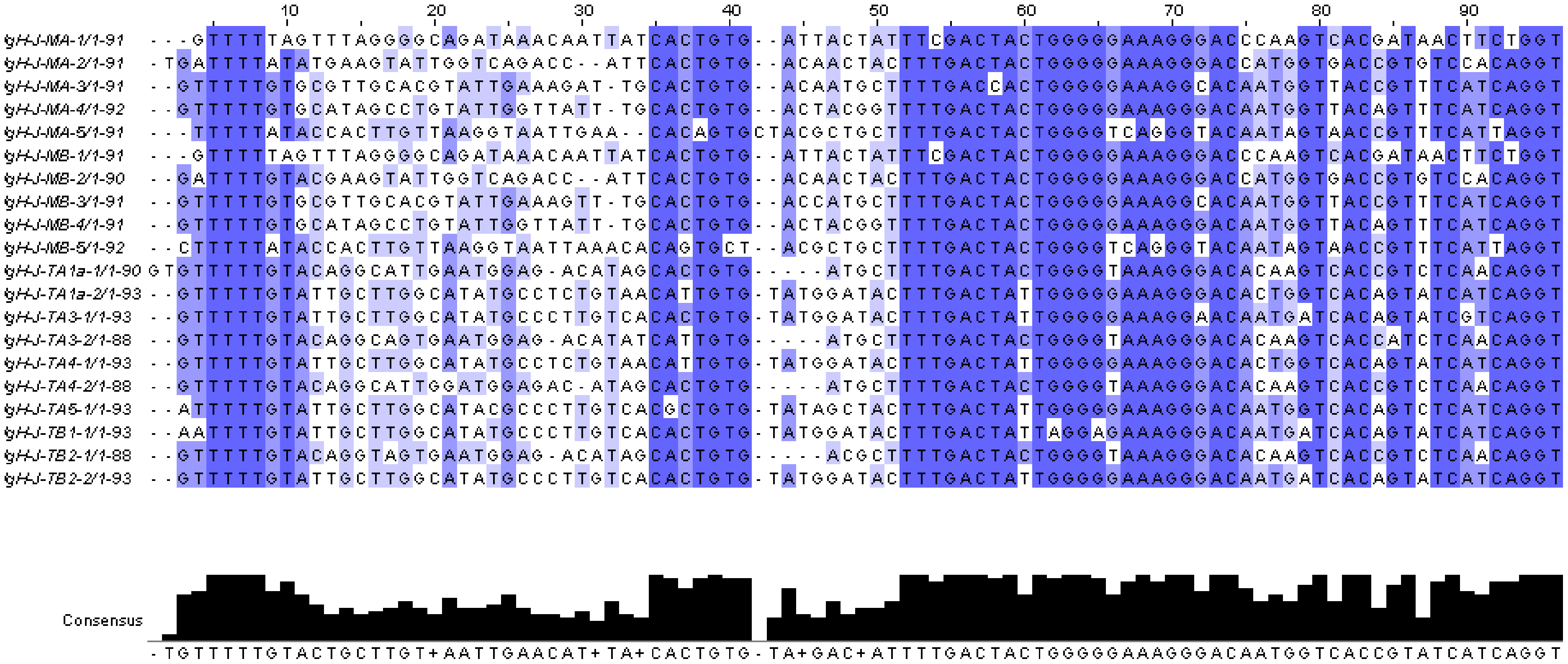
10 20 30 40 50 60 70 80 90
gHJ-MA-1/1-91 ---GTTTTTAGTTTAGGGGCAGATAAACAATTATCACTGTG--ATTACTATTTCGACTACTGGGGGAAAGGGACCCAAGTCACGATAACTTCTGGT
gHJ-MA-2/1-91 -TGATTTTATATGAAGTATTGGTCAGACC--ATTCACTGTG--ACAACTACTTTGACTACTGGGGGAAAGGGACCATGGTGACCGTGTCCACAGGT
gHJ-MA-3/1-91 --GTTTTTGTGCGTTGCACGTATTGAAAGAT-TGCACTGTG--ACAATGCTTTTGACCACTGGGGGAAAGGCACAATGGTTACCGTTTCATCAGGT
gHJ-MA-4/1-92 --GTTTTTGTGCATAGCCTGTATTGGTTATT-TGCACTGTG--ACTACGGTTTTGACTACTGGGGGAAAGGGACAATGGTTACAGTTTCATCAGGT
gHJ-MA-5/1-91 ---TTTTTATACCACTTGTTAAGGTAATTGAA--CACAGTGCTACGCTGCTTTTGACTACTGGGGTCAGGGTACAATAGTAACCGTTTCATTAGGT
gHJ-MB-1/1-91 ---GTTTTTAGTTTAGGGGCAGATAAACAATTATCACTGTG--ATTACTATTTCGACTACTGGGGGAAAGGGACCCAAGTCACGATAACTTCTGGT
gHJ-MB-2/1-90 --GATTTTGTACGAAGTATTGGTCAGACC--ATTCACTGTG--ACAACTACTTTGACTACTGGGGGAAAGGGACCATGGTGACCGTGTCCACAGGT
gHJ-MB-3/1-91 --GTTTTTGTGCGTTGCACGTATTGAAAGTT-TGCACTGTG--ACCATGCTTTTGACTACTGGGGGAAAGGCACAATGGTTACCGTTTCATCAGGT
gHJ-MB-4/1-91 --GTTTTTGTGCATAGCCTGTATTGGTTATT-TGCACTGTG--ACTACGGTTTTGACTACTGGGGGAAAGGGACAATGGTTACAGTTTCATCAGGT
gHJ-MB-5/1-92 --CTTTTTATACCACTTGTTAAGGTAATTAAACACAGTGCT--ACGCTGCTTTTGACTACTGGGGTCAGGGTACAATAGTAACCGTTTCATTAGGT
gHJ-TA1a-1/1-90 GTGTTTTTGTACAGGCATTGAATGGAG-ACATAGCACTGTG----ATGCTTTTGACTACTGGGGTAAAGGGACACAAGTCACCGTCTCAACAGGT
gHJ-TA1a-2/1-93 --GTTTTTGTATTGCTTGGCATATGCCTCTGTAACATTGTG-TATGGATACTTTGACTATTGGGGGAAAGGGACACTGGTCACAGTATCATCAGGT
gHJ-TA3-1/1-93 --GTTTTTGTATTGCTTGGCATATGCCCTTGTCACACTGTG-TATGGATACTTTGACTATTGGGGGAAAGGAACAATGATCACAGTATCGTCAGGT
gHJ-TA3-2/1-88 --GTTTTTGTACAGGCAGTGAATGGAG-ACATATCATTGTG----ATGCTTTTGACTACTGGGGTAAAGGGACACAAGTCACCATCTCAACAGGT
gHJ-TA4-1/1-93 --GTTTTTGTATTGCTTGGCATATGCCTCTGTAACATTGTG-TATGGATACTTTGACTATTGGGGGAAAGGGACACTGGTCACAGTATCATCAGGT
gHJ-TA4-2/1-88 --GTTTTTGTACAGGCATTGGATGGAGAC-ATAGCACTGTG----ATGCTTTTGACTACTGGGGTAAAGGGACACAAGTCACCGTCTCAACAGGT
gHJ-TA5-1/1-93 --ATTTTTGTATTGCTTGGCATACGCCCTTGTCACGCTGTG-TATAGCTACTTTGACTATTGGGGGAAAGGGACAATGGTCACAGTCTCATCAGGT
gHJ-TB1-1/1-93 --AATTTTGTATTGCTTGGCATATGCCCTTGTCACACTGTG-TATGGATACTTTGACTATTAGGAGAAAGGGACAATGATCACAGTATCATCAGGT
gHJ-TB2-1/1-88 --GTTTTTGTACAGGTAGTGAATGGAG-ACATAGCACTGTG----ACGCTTTTGACTACTGGGGTAAAGGGACACAAGTCACCGTCTCAACAGGT
gHJ-TB2-2/1-93 --GTTTTTGTATTGCTTGGCATATGCCCTTGTCACACTGTG-TATGGATACTTTGACTATTGGGGGAAAGGGACAATGATCACAGTATCATCAGGT
Consensus
-TGTTTTTGTACTGCTTGT+AATTGAACAT+TA+CACTGTG-TA+GAC+ATTTTGACTACTGGGGGAAAGGGACAATGGTCACCGTATCATCAGGT
