# Supplementary material for: Evolution of duplicated IgH loci in Atlantic salmon, Salmo salar
Source: BMC Genomics. 2010 Sep 2;11:486. doi: 10.1186/1471-2164-11-486 (PMC2996982; doi:10.1186/1471-2164-11-486)
Supplement: Additional file 11 — Alignment of amino acid VH sequences. This file contains a multiple sequence alignment of amino acid VH sequences obtained from ClustalW. Identical residues are shown as dots (.) and gaps are shown as hyphens (-). [file 1471-2164-11-486-S11.PDF]

## $V_H$ genes

```
                                   FR1                    CDR1        FR2         CDR2                     FR3
IGHBV1-01  ----VHSQT- LTESGPVVK- KPGESHQLTC TASGFTFSS- ---YWMAWIR QAPGKGPEWI ASS--YSTT- TYYSQSVQGR FTISRDDSSS KLYLQMSSLK SEDTAVYYCA --
IGHBV1-02  ----.YG..- .........- .......... .........- ---SR.G... ......L... .YISTQ.NP- IS........ .......... .....LN... .......... --
IGHVA1-05  ---G..G..- .........- ......K... .........- ---.G.N... ......L... .Y.--H...- V......... .......... ......N... .........G --
IGHBV1-06  ----.YG..- .........- ......K... .......T.- ---.Y.G... ..K..EF... GHI--HGG.- ......F... .......... ......N... .......... --
IGHVA1-07  ----..G..- .........- ......K... .........- ---.E.S... ......L... .Y.--...N- .......... .......... ......N... .......... --
IGHVB1-07  ----.YG..- .........- ......K... .........- ---.Y.G... ..K..EF... DHI--HGG.- ......F... ..L..E.... ......N.P. .......... --
IGHVA1-08  ----..G..- .........- ......K... ........I- ---.Y..... ......L.LV .YIGTS.SP- IS........ .......... ......N... .......... --
IGHBV1-09  ----FQG.R- .I.......- ..E.P.K... I...LDMNN- ---.R..... ......L..V .AITHD.RS- ...P...... ......N.MK QV....N... T..S...... --
IGHVA1-10  ---GIQG..- ..D......- .TE...K... .GA......- ---....... ......L... GII--HG..- .......... ..L....... ......N... .......... --
IGHBV1-13  ----.QG..- .........- ..Q...K... .........- ---....... ......L..V SYISSDGGS- ...P...... ......N.KQ QV....N... T..S...... --
IGHBV1-14  ----.QG..- .........- ......K... ...DLDVN.- ---....... ......L..V SDIRKDGGS- .........Q ......N.KQ QV....N... T..S...... --
IGHAV1-14  ISTG..G..- .........- .......... .G.......- ---..I.... ......L.F. .AH-YN.IN- IA........ ........KQ QV....N..N T..S..---- --
IGHBV8-03  ----..C.VV ..QAEQS.QG T.AG.LK... AC....L..- ---TN.Y... ..T...L... IYY-YSEGS- KSNA.V.... ..A.K.--.. NF..H.NL.. ...S...... --
IGHBV8-02  ----..C.VV ..QAEQS.QG T.KG.IK... AC....L..- ---TN.Y... ..T...L... IYY-YSDSD- KSNA.V.... ..A.K.--.T NF..H..Q.. P..S...... --
IGHVA8-02  ---G..C.VV ..QAEQT.QG T..G.LK... AC..I.L..- ---SY.H... ......L... IYY-YSD.Y- KSNAPV.... ..A.K.--.T NF..H..Q.. ...S...... --
IGHVA8-01  ---G..C.VV ..QAEQT.QG T..G.LK... AC..I.L..- ---SY.H... ......L... IYY-YSD.Y- KSNAPV.... ..A.K.--.T NF..H..Q.. ...S...... --
IGHVA8-03  ---G..C.VV ..QAEQS.QG T..G.LK... AC..I.L.N- ---TN.H... ......L... IYY-YSD.Y- KSNAPV.... ..A.K.--.. NF..H..Q.. ...S...... --
IGHVA8-05  ---G..C.VV ..QAEQS.QG T.AG.LK... AC....L..- ---TR.H... ......L... IYY-YSD.Y- KSNAPV.... ..A.K.--.T NF..H..Q.. P..S...... --
IGHVA8-08  ----..C.VV ..QAEQS.QG T.AG.LK... AC..I.L..- ---SY.N... ......L... MFY-YSDSS- KSNAPV.... ..A.K.--.T NF..H..Q.N P..S...... --
IGHVA8-07  ----..C.VV ..QAEQS.QG T.AG.LK... AC..I.L..- ---SY.N... ......L... MFY-YSDSS- KSNAPV.... ..A.K.--.T NF..H..Q.N P..S...... --
IGHVA8-13  ---G..C.VV ..QAEQS.QG TS.G.LK... AC....L..- ---.R.H... ....Q.LQ.. LHY-YS.SD- NG.TLVL... ..A.K.--.T NF..H.TQ.. P..S...... --
IGHVA8-11  ----..C.VV ..QAEQS.QG TS.G.LK... AC..I.L..- ---.R.H..H P...Q.LQ.. FHY-YS.SD- NG.TLVL... .IA.K.--.T NF..H.TQ.. P..S....S. --
IGHVA8-12  ---G..C.VV ..QAEQS.QG TS.G.LK... AC....L..- ---.R.H... ....Q.LQ.. LHY-YT.SD- NG.ALVL... ..A.K.--.A NF..H.TQ.. P..S...... --
IGHBV4-04  ---------- -------..- R..DTVK.S. .I...SM.G- ---..IH.M. .NQ..AL... GRMNSN.--- P...D.LKDQ ..LTE.V.T. TQF.EAK..R ...S...... --
IGHBV4-03  ---------- -------L.- R.EDTVK.S. KT....MT.- ---FY.Y... .E...AL... GRMNSN.--- PD..D.LK.Q .ILTE.V.T. TQF.EAK..R ...S.....- --
IGHBV4-09  ---------R .DQ.PSQ..- I..H.VKVS. II..YSMT.- ---HNIH... .K....L... GRMNTG.G.D VI.AD.LK.Q ..LTE.V.T. TQF.EAK..S ...S...... --
IGHBV4-08  ---------- -------..- I..H.VKVS. II..YSMT.- ---HNIH... .K....L... GRMNTG.G.D VI.AD.LK.Q ..LTE.V.T. TQF.EAK..S ...S...... --
IGHVA4-08  ----.CCDIR .DQ.PSQ..- I..H.VKVS. II..YSMT.- ---NNIH... .K....L... GRMYTG.G.D VI.AD.LK.Q ..LTE.V.T. TQF.EAK..I ...S...... --
IGHBV9-01  --VL.VQGQS ..S.E....- .....VT.S. .V...SMG.- ---.N.H... .K....L... GRIDTGTG-- .IFA..L..Q ...TK.N.KN Q...EVK... T..S...... --
IGHBV6-13  ------V.SY ELTQPASMTV Q..QPLTIS. KV.-YSVT.- ---LYT.... .PA..TL... GYI--S.GGS .A.KD.LKNK .S.T..T..N TVF.KGQ..Q T......... --
IGHVA6-10  ------V.SY ELTQPASMTV Q..QPLTIS. KV.-YSVT.- ---LHT.... .PA..TL..F GYI--H.GGS M..KD.LKNK .S.T.ET..N T.F.KGQ..Q T......... --
IGHBV6-15  ---------E ELTQPAFMTV QS.QLLTIS. KV.-YSVT.- ---VATF... .PA..TL... GYI--NGGGS .A.KD.LKNK .S.T..T..N T.F.KGQ..Q T......... --
IGHBV6-14  ---------E ELTQPAFMTV QS.QLLTIS. KV.-YSVT.- ---VATF... .PA..TL... GYI--N.GGS .A.KD.LKNK .S.T..T..N T.F.KGQ..Q T......... --
IGHBV6-08  ------V.SY ELSQPTSMTV Q..QPLTIS. KV.-YSVTD- ---T.TS... .PA..AL... GNV--..G-D .K.KD.LKNK .SL.V.S..N TVF.KGQN.Q T......... --
IGHBV6-07  ------V.SY ELSQPTSMTV Q..QPLTIS. KV.-YSVTN- ---T.TS... .PA..AL... GNV--..G-D .K.KD.LKNK .SL.V.S..N TVF.KGQN.Q T......... --
IGHVA6-02  ------V.SN ELNQPASMTV Q..QPLTIS. KV.-YSVTD- ---T.T.... .PA..TL... GNV--..G-D .E.KD.LKNK .SL.V.S..N TVF.KGQN.Q T......... --
IGHVA6-04  -----SV.SY ELSQPASMTV Q..QPLTIS. KV.-YYVT.- ---LHT.... .PA..TL... GNV--..G-D .K.NDPLKNK .SV.V.S..N TVF.KG-NFQ T......... --
IGHBV6-06  -------.CE ELTQPASMTV Q..QPLTIS. KV.-YSVG.- ---.YT.... .PA...L... GMK--.TG-G .HHKD.LKNK .SLTL.S..N TVT.TGQN.Q A......... --
IGHVA3-01  -----VFCQT ELTQPGSMIL Q..QPLT.S. KV..YSLT.S --S.CTG.V. .HA..AL..V GYI--C.SGN I...DKLKNK .S....T..N TVF.KGQ..Q T......... --
IGHVA7-03  ---YCG--GL ELSQPS.MVI .....PSI.. KV..YSV.DS SISFATG.V. KPA..AM... SHI--WYDGD ILKNYALKNK ......A..N SVS..GQ..Q P.......V --
IGHVA7-02  ---------- -LSQPS.MVV .....PSF.. KV..YSV.DS SISFATG.V. KPA..SM... SHI--WDDGD I.KNDALKNK .S....A..N SVS..GQ..Q P.......V --
IGHVA17-01 ---VCGIVDP ELTQPISMVV .....LSI.. KV..YSI.DD --S.TTD..G HPVD.AM... G------DSD GNCKD.LKS. .S..K...YN TVT.EGQRRL I......D.V --
IGHBV12-01 --VHCG---L EFTQPSSLDV .....LSI.. KV..YSLTDS TNLHGIG... HPT..AL... WTI--.YDGS SNSQS.LKD. .SFT..T..N TVF.KGQ..Q T......... --
IGHBV10-04 -----GYCGI ELIHSGP.VI .R...FSNS. KF...SIY.- ---.CPHC.Q ..E..VLKHV GYN--CTS-S ..TVD.LNSK ISF.Y.SF.. TVF..GNN.Q T....E...V --
IGHBV2-01  ----CNG.SL EVIPSSP.QK ....TLS.S. KI..Y.VT.- ---HHTH... .PA..SL..M G---FSGLGL G.HAKKFE.. METTK.TRN. M.T.ML.GVR A..S...... --
IGHBV2-05  ----LNG.SL ESIPSSP.LK ....TLS.S. KI..Y..D.- ---HHTH... .PA..SL..M G---YSGLGL G.HAKRFE.. METTKAT.N. M.T.KL.G.R A..S...... --
IGHBV2-06  ----LKG.SL ESSPSSS.MK .L..TIS.S. KI.....N.- ---HNTH... .PA..SL..M G---YSGLGL G.HAKTFE.. METTK.N.N. MMT.KL.G.R A..S...... --
IGHBV15-03 ----LNG.SM ESIPSSP.MK ...DTLN.S. KG..Y..GN- ---HAVH.V. .PK...L... GRV-RTD.GE AT.AN.IS.. LE.TK.NAQ. MSF.K..G.R A..S...... --
IGHVA15-01 ------G..M ESIPSSPLQK ....TLN.S. RGT.Y..T.- ---.G.N... .PT..PL..M GWI-NTN.GA AG.AKTLE.. IELTK.S.V. MTH.KL.G.. A..S...... RW
IGHBV16-11 ----LNG.SM ESIPSSPLRK ....TLS.S. KG.WY.QCEK ---.G.S... .PA..SL... G-----Y.GT --.TK..E.. IE.T..NTIC IV..KL.V.R A..F...H.. --
IGHBV16-13 ----LNG.SM ESIPSSPLQK ....TLSHS. KG..Y.QCEK ---.G.S... .PA..SL... G-----Y.GK --.TK..E.. IE.T..NTIC MV..KL.V.R A..S...... --
IGHBV16-07 ----LNG.SM ESIPSGP.LK ...DTLS.S. KG.S....N- ---HG.H... .PA..ALD.M GII-Y-YASK .I.L.NIE.. IEVT..N.KG MVN.KL.G.R A..S...... --
IGHBV16-04 ----.YG.SL EAIPSSP.LK N...PVS.S. KG.....G.- ---.H.G... .PA..AL... GII-WNDASG .A.AK..E.. IEVT..N.N. MV..KL.G.R A..S...... --
```
