# Supplementary material for: Evolution of duplicated IgH loci in Atlantic salmon, Salmo salar
Source: BMC Genomics. 2010 Sep 2;11:486. doi: 10.1186/1471-2164-11-486 (PMC2996982; doi:10.1186/1471-2164-11-486)
Supplement: Additional file 12 — Distribution of variable (VH) families in the two IgH loci. Table showing the number of sequences identified per family. [file 1471-2164-11-486-S12.PDF]

**Family**	***IGH-A***	***IGH-B***
1	17	18
2	2	7
3	2	2
4	12	12
5	1	2
6	13	18
7	3	1
8	15	6
9	2	7
10	11	4
11	1	5
12	1	1
13	1	0
14	2	0
15	5	4
16	6	13
17	4	3
18	1	0
