# Supplementary material for: Evolution of duplicated IgH loci in Atlantic salmon, Salmo salar
Source: BMC Genomics. 2010 Sep 2;11:486. doi: 10.1186/1471-2164-11-486 (PMC2996982; doi:10.1186/1471-2164-11-486)
Supplement: Additional file 13 — PCR primers and oligo probes. Table listing the PCR primers and oligo probes used in this study. [file 1471-2164-11-486-S13.PDF]

| Discription | Name | Primer sequence |
| --- | --- | --- |
| **Expression study variable region** | vp-001 | AAAACAAGTTCAGCATCACCAG |
| | vp-002 | AAACTGTCTGGTCTGACTCTGC |
| | vp-003 | AACAGTCTGAAGAGTGAGGACACA |
| | vp-004 | ACAACTTACAGACTGAAGACACAGC |
| | vp-005 | ACAAGTTCAGCATCACCAGAG |
| | vp-006 | ACATCTCCAAAAACCAGCTGTA |
| | vp-007 | ACATGTTTTTAAAAGGGCAGAAC |
| | vp-008 | ACGGACAGAAGTCACCAGAGAG |
| | vp-009 | ACGTTTCAGTAATTCAGTGCCTTT |
| | vp-010 | ACTCTGAATCTGTCTGGTCTGAG |
| | vp-011 | ACTTACAGATGAACAGCCTGAAGA |
| | vp-012 | AGACAACAGCAAGAGGCAGGT |
| | vp-013 | AGACTGAAGACTCTGCTGTGAAT |
| | vp-014 | AGAGCAGATTCCCCATCTCC |
| | vp-015 | AGATGCCTCCAGTAACTCTGTG |
| | vp-016 | AGCAGCAGGTGTATCTCCAAA |
| | vp-017 | AGCATGATGACTCTGAAGCTGT |
| | vp-018 | AGCCCGAAGAGTGAGGACAC |
| | vp-019 | AGCCCTGAAGACTGAAGACTTTG |
| | vp-020 | AGCTTTGGAGTATGTTGGTTGTA |
| | vp-021 | AGGACGTGTCCACCAGCAC |
| | vp-022 | AGGCCAAGATTCTGAGAGTAGAG |
| | vp-023 | AGGGACGGATTGAACTGACC |
| | vp-024 | AGGTGTATCTCCAAATGAACAGC |
| | vp-025 | ATCCCTCCAGCAACTCTGTG |
| | vp-026 | ATCTACACATGAGCCAGTTGAA |
| | vp-027 | ATCTCCAGAGACACTTCCAGCAA |
| | vp-028 | ATGCTGTCTGGTGTGAGAGC |
| | vp-029 | ATTCACCATCTCCAGAGACGAC |
| | vp-030 | ATTGTGCCCGAGAGACACAG |
| | vp-031 | ATTGTGCTCGGTGGTCACAGT |
| | vp-032 | CAACAACCTGCAGGGAAAG |
| | vp-033 | CAACAGCATGTTGACTTTGAA |
| | vp-034 | CAACTCTAACAGGACAGAACCTG |
| | vp-035 | CAAGAGAGACGCATTTGTGTG |
| | vp-036 | CAAGGACGATTTATAGCATCGAA |
| | vp-037 | CAGAGATGACTCAAGCAGTAAGC |
| | vp-038 | CAGATGAACAGCTTGAAGACTGAA |
| | vp-039 | CAGCACCAGCAGTACAGTGTTT |
| | vp-040 | CAGCCTGTCAGTAGACTCTAGCA |
| | vp-041 | CAGCTCCAAACACCAGCTGTA |
| | vp-042 | CAGGGCCAGTTCACCATC |
| | vp-043 | CAGTGAGCATGACACATCTGAAA |
| | vp-044 | CAGTTCTTAGGGGCCAAGATT |
| | vp-045 | CATCACCAGAGATACTTCCAGCA |
| | vp-046 | CATCGAAGGACGCTCTAATTT |
| | vp-047 | CATCTCCAAAGATGGATCCAAAA |
| | vp-048 | CATCTCCAGAGACAACAGCAAA |
| | vp-049 | CATTTTGAGAGGCACAGTGG |
| | vp-050 | CCAAAGACACAGTGACACAGGA |
| | vp-051 | CCAGAGAAATGTACAGCAGCA |
| | vp-052 | CCATTTCCAGAGATGACTCCA |
| | vp-053 | CCCGGTACACACAGTGAGAG |
| | vp-054 | CCGAGAAGTCAAACTGAAGACA |
| | vp-055 | CCGAGATAATACCATCTGCATTG |
| | vp-056 | CCGGTACACACAGTGAGTGAG |
| | vp-057 | CCTACAGATGAGCAGCCTGAA |
| | vp-058 | CCTCACAGTGATATAAACCACCA |
| | vp-059 | CGAAGGATAGCGCTAATTTCTA |
| | vp-060 | CGACAGACACACTGATTGAAGC |
| | vp-061 | CGACAGCCTGAAGGAAAAGC |
| | vp-062 | CGTCTCCACAAGTACCCAGTTC |
| | vp-063 | CTCCACAAGCACCCAGTTCTTA |
| | vp-064 | CTCCAGAGACATTAGCATGACAC |
| **Expression study variable region** | vp-090 | GCCAGTTCACTCTGACTGTGG |
| | vp-091 | GCTGAAGGTAAAGCTTTGGTG |
| | vp-092 | GGAAAACATTGGAGTGGATTG |
| | vp-093 | GGAGGCACAGTGCAACAG |
| | vp-094 | GGCACCACCAAGTCATTACAA |
| | vp-095 | GGCGTATAGAAGTCACCAGAGA |
| | vp-096 | GTCAGAGTAAGAACTCAACGGACA |
| | vp-097 | GTCAGTTCACCATCACCAAAGA |
| | vp-098 | GTCCTCGCTGAATACGACAAG |
| | vp-099 | GTCTTCACTGGATACGACAAGC |
| | vp-100 | GTGCTCAAAGACCACAGTGA |
| | vp-101 | TAAATTAAATAGAACAGAGATGCTTCA |
| | vp-102 | TATTGTGCCCGAGACAGTGA |
| | vp-103 | TCAGCCTCTCAGTAGAATCTAGCA |
| | vp-104 | TCAGCCTGTCAGTAGACTCAAGC |
| | vp-105 | TCTAAAGGCATGGTTAATTTGAA |
| | vp-106 | TCTCCACCAGCACCCACTT |
| | vp-107 | TCTTAATGGCCAGAAGTCTGAGAT |
| | vp-108 | TGAACAGCCTGAAGAGTGAAGA |
| | vp-109 | TGATGGTGACAGCACTTACTACC |
| | vp-110 | TGCAAGAAAGACACAGTGGTG |
| | vp-111 | TGCACGAGAGACACAATGG |
| | vp-112 | TGGCCACAGTGATACAAACCA |
| | vp-113 | TGTGCTCGAGAGACACACTGA |
| | vp-114 | TTATTACCCTGCTCGACAGACA |
| | vp-115 | CTGGAATGGGTTGCTTTTATTA |
| | vp-116 | CTACAGATGAACAGCCTGAAGA |
| | vp-117 | AGGTGTATCTCCAAATGAACAGC |
| | vp-118 | AGTCCAGGGTCGGTTGAC |
| | vp-119 | CATCTCCAGAGACAACAGCAAA |
| | vp-120 | GATAAGACAACCTGCAGGGAAA |
| | vp-121 | GCTGTCTGATCTGAGAGAAGAGGAC |
| | vp-122 | CACTTAGACATCACCAGTCTGC |
| | vp-123 | TTAAATGCTTTGTCCACAGGTA |
| | vp-124 | GAAGGCAAAGCTCTGGAACAT |
| | vp-125 | AGGCCAAGAGTCTAAGTTCAGAGG |
| | vp-126 | AGTGAGATCGTCTCCATAAACACA |
| | vp-127 | ATTATTGTCCTCGGTGAACACAGT |
| | vp-128 | ATTTACAGCATCGAAGGATAGCA |
| | vp-129 | CAGGCACATTGGTGTGAGT |
| | vp-130 | CAGGCTGTGTATTATTGTGCTC |
| | vp-131 | CCAGGAGTAGGCTGTAAGCTGT |
| | vp-132 | CCTATTGTGCCCAAGTCTCAC |
| | vp-133 | CTCTGCTGTCTATTACTGTGCAAGA |
| | vp-134 | CTGATGACGTCTCCATAAACACA |
| | vp-135 | CTGCATGGTGTATCTGAAGCTGT |
| | vp-136 | CTGTGCACGCTGGGTACAGT |
| | vp-137 | CTGTGGCCGGTACACACAGT |
| | vp-138 | CTGTGTATTACTGGGCATGCTG |
| | vp-139 | GAGACAATAGCATGAAACAGGTG |
| | vp-140 | GAGATGACAGCAAGCAGCAG |
| | vp-141 | GAGGACACGGCAGTGTATTACT |
| | vp-142 | TATTATTGTGCTCGGGGGTCA |
| | vp-143 | TCACAGCATCGAAGGACAGC |
| | vp-144 | TCGAAGGACAGCGCTAATTT |
| | vp-145 | AAAACAGCATCACATTGTTTTCA |
| | vp-146 | CAGCCTCAATTTAGACTCCTCTTT |
| | vp-147 | ACAGGGTAGAACCTACAGAGTGAAG |
| | vp-148 | ACCGTGCTTTTTGAAGGGAAC |
| | vp-149 | AGAAGTCTAACTGAAGACACAGCACT |
| | vp-150 | AGTATTACTGTGCTCGCCTCCA |
| | vp-151 | AGTCTGTGAGACACAGTGATATAAACC |
| | vp-152 | AGTACAGCGTTTCTACAAGGGAAC |
| | vp-153 | ATTATTGTCCTCGGTGAACACAGT |

(Continued on next page)

| | | | | | |
|---|---|---|---|---|---|
| | vp-065 | CTCGGTGGACACAGTGATACAA | | vp-154 | CAACAGTTTGAAGAGTGAAGACACA |
| | vp-066 | CTGCAGTTTAGGTGGGGATG | | vp-155 | GTCAGTTGAAGCCAGAGGACT |
| | vp-067 | CTGCTGCACATGAGCCAGTT | | vp-156 | GAGATGACAGCAAGCAGCAG |
| | vp-068 | CTGGTCTGACGCTGCTGTG | | vp-157 | CAGGGGTAGTTCACCATCACC |
| | vp-069 | CTGTAACTTTAACAGGGCAGAGTC | | vp-158 | CACCAGAGACAATTCTAACAGCAT |
| | vp-070 | CTGTGCCAGAGACACAGTGA | | vp-159 | CAAGAAAGACAGTGCTGTGAGTG |
| | vp-071 | CTGTGCCTACCCTCACAGTGATA | | vp-160 | GCACGAGAGACACAGTGGTG |
| | vp-072 | CTTCCACTGTAGACTCACTAAAGAGC | | vp-161 | CATGACTCTGAAGCTGTCTGG |
| | vp-073 | CTTGAGAGGCAATGTGACTGAA | | vp-162 | AGCAACACTTCCAGCAACAT |
| | vp-074 | CTTTAACAGGGCAGAACCTG | | vp-163 | AAAACAAGTTCAGCACCACCA |
| | vp-075 | GAAAGCATTGGAATGGATAGTTT | | vp-164 | AAAGCTTTGGCGCATGTTG |
| | vp-076 | GACACTTTGAAAGGCACAGTGA | | vp-165 | CCGATTCACTGCATCAAAGG |
| | vp-077 | GACTATAACAGGGCAGAACCTG | | vp-166 | GCCAGTTCATTCTGACTGAGG |
| | vp-078 | GAGAGACTTCCAGCAACACG | | vp-167 | GCCCAAAGCATGTCATTCC |
| | vp-079 | GAGCTAAATGAAAACAGGCAAGA | | vp-168 | GAGCCAGTTGAATCCAGAGGA |
| | vp-080 | GAGGAAAATTCCAACATCTTCA | | vp-169 | CACTGTAGATTCACTGAACAGCAAG |
| | vp-081 | GAGGCCAAGAGTCTCAGATCA | | vp-170 | AACAGGATGATGACTTTGATGCT |
| | vp-082 | GATAACTCAAACTGAAGACACAGC | | vp-171 | TACCCTCACAGTGATACAAACCA |
| | vp-083 | GATGGACAGAAGTCACCAGAGAG | | vp-172 | GAACCTGTTGAAGTCAGAGGAC |
| | vp-084 | GATTCTCCAGAGATGACTCCAG | | vp-173 | TCAGTTTCACAAGAGACACTTCCA |
| | vp-085 | GCAAGCAGCAGGTTTATCTCC | | vp-174 | ACGATTCACTGCATCAAAGGA |
| | vp-086 | GCAGTCGAGAGACACACTGA | | vp-175 | GGCTAATTGAAGACACAGCAG |
| | vp-087 | GCATGACTCATCTGAAACTGTCT | | vp-176 | ATTGGGATAATCTGAGGTGGTG |
| | vp-088 | GCATTTAGAGGTGAAAAGCCTAA | | vp-177 | GACACAGTGACTGGAGCTGGT |
| **Expression study** | vp-089 | GCATTTCAAGAGATGCTTCCA | | vp-178 | AGATTCACTGCATCGAAGGAC |
| **Constant region** | | | **RT-PCR** | | |
| Cμ outer set | cmAB-2 | TCATTGGCAAAGCAGGCGAAG | Cμ | Cm1F | ACTCCGTGTAAAGAGAGCAG |
| inner set | cmAp-2 | GACGGGTGCTGCAGATATTCCG | | Cm1R | AAGCATATCCTCAAGAGACG |
| | cmBp-2 | GACGGGTGCTGCAGATATTCCA | Cδ | Cd1F | TTGTGTCTTGTGAGGTGAAG |
| Cδ outer set | cdAB-1 | AGCTGTTGGAGTAGCCTTGG | | Cd1R | GGTGATGTTGATGTCCGAAG |
| inner set | cdAp-2 | TTGGAGGCAGGCAGATCT | CτB-2 | CtB2F | TCGTCCAATACCCGGCAGTC |
| | cdBp-2 | TTGGAGGCAGGCAGATCA | | CtB2R | TCATCAGTCTTGGCCTGTTC |
| Cτ outer set | ctO-1 | ATGACTGCTGTCTTTCCACCT | CτA-3 | CtA3F | TGTCCAGTACCCGTTGTTTC |
| | ctO-2 | TTGATGACTGCTGTCTTTACAACT | | CtA3R | CCATCAGTCTTGGCCTATTC |
| inner set | ctBp-1 | ACGTTGCAGTATCCCAGACA | CτA-4 | CtA4F | TGTCCAGTACCCTTCGGTTC |
| | ctAp-2 | CCAGACACTCTTAGCCACTCG | | CtA4R | CCATCCATCTTGTCCTCTCC |
| | ctAp-3 | TGAGTTTTCCCAGACATTAGCC | CτA-5 | CtA5F | TCGTCCAGTACCCAGCAGTC |
| | ctAp-4 | TTCCCAGTCATTCTTAGACACG | | CtA5R | TGATCCGTCTGTTTCCGTCC |
| **Probe_PCR** | | | | IL1bF*[1] | CCCATCACCATGCGTCAC |
| Cμ exon 4 | CμF | GAGATCCACAGCGTCCATCT | | IL1bR*[1] | CTCCAACTCCAACACTATATG |
| | CμR | GTGGACTTGATCATGCTTTC | | TNFa2F*[1] | TGCTGGCAATGCAAAAGTAG |
| Cδ exon 7 | CδF | TCAGCCCTTCGGACATCAAC | | TNFa2R*[1] | AGCCTGGCTGTAAACGAAGA |
| | CδR | CTGGTTTGGATATGTTCAGG | | COX2F*[1] | ATCCTTACTCACTACAAAGG |
| Cτ exon 4 | CτF | TTCTGTGTCCGTCCACCTTCT | | COXR*[1] | GCTGGTCCTTTCATGAAGTCTG |
| | CτR | TTGTCCAAGCCGGCGTGCTT | | Mx3F*[2] | TGAGGACTCGGCAGAAAGGATGTA |
| | | | | Mx3R*[2] | CTTCGCGGATTTCAGGAGGTTAGG |
| **Oligo probe** | Cμ | CTAGCCAGATTCAATCAGGAAGGACCTACTCTGTCTACAGTCAGCTCACATTTAGCAATGACTTGTGGAA | | | |
| | Cδ | TGAGCTCGTCTGCCTGGTGCTTGGCTTCAGCCCTTCGGACATCAACATCACCTGGCTGCTGGACAACGTC | | | |
| | Cτ | GACTCTGGTGTGCCTGGTGGTCTGCCCATCTCTATGTGACGTCTACATCATGTGGCAGGTGAACAG | | | |

## References


*[1] IL1bF, IL1bR, TNFa2F, TNFa2R, COX2F and COX2R

Ingerslev, H. C., C. Cunningham, and H. I. Wergeland: **Cloning and expression of TNF-α, IL-1β and COX-2 in an anadromous and landlocked strain of Atlantic salmon (*Salmo salar* L.) during the smolting period.** *Fish Shellfish Immunol* 2006, **20**:450-461.

*[2] Mx3F and Mx3R

Lockhart, K., S. K. Gahlawat, D. Soto-Mosquera, T. J. Bowden, and A. E. Ellis: **IPNV carrier Atlantic salmon growers do not express Mx mRNA and poly I:C-induced Mx response does not cure the carrier state.** *Fish Shellfish Immunol* 2004, **17**:347-352.
